# Simple form of a projection set in hybrid iterative schemes for non-linear mappings, application of inequalities and computational experiments

**DOI:** 10.1186/s13660-018-1774-z

**Published:** 2018-07-17

**Authors:** Li Wei, Ravi P. Agarwal

**Affiliations:** 1 School of Mathematics and Statistics, Hebei University of Economics and Business, Shijiazhuang, China; 2 Department of Mathematics, Texas A&M University—Kingsville, Kingsville, USA; 3 Florida Institute of Technology, Melbourne, USA

**Keywords:** Iterative scheme, Lyapunov functional, Metric projection, Maximal monotone operator, Weakly relatively non-expansive mapping

## Abstract

Some relaxed hybrid iterative schemes for approximating a common element of the sets of zeros of infinite maximal monotone operators and the sets of fixed points of infinite weakly relatively non-expansive mappings in a real Banach space are presented. Under mild assumptions, some strong convergence theorems are proved. Compared to recent work, two new projection sets are constructed, which avoids calculating infinite projection sets for each iterative step. Some inequalities are employed sufficiently to show the convergence of the iterative sequences. A specific example is listed to test the effectiveness of the new iterative schemes, and computational experiments are conducted. From the example, we can see that although we have infinite choices to choose the iterative sequences from an interval, different choice corresponds to different rate of convergence.

## Introduction

Throughout this paper, let *X* be a real Banach space with norm $\|\cdot \|$ and $X^{*}$ be the dual space of *X*. Let *K* be a non-empty closed and convex subset of *X*. Let $\langle x, f\rangle$ be the value of $f \in X^{*}$ at $x \in X$. We write $x_{n} \rightarrow x$ to denote that $\{ x_{n}\}$ converges strongly to *x* and $x_{n} \rightharpoonup x$ to denote that $\{x_{n}\}$ converges weakly to *x*.

Suppose that *A* is a multi-valued operator from *X* into $X^{*}$. *A* is said to be monotone [1] if for $\forall v_{i} \in Au_{i}$, $i = 1,2$, one has $\langle u_{1} - u_{2}, v_{1} - v_{2}\rangle\geq0$. The monotone operator *A* is called maximal monotone if $R(J+k A) = X^{*}$, for $k > 0$, where $J: X \rightarrow2^{X^{*}}$ is the normalized duality mapping defined by

$$J(x) = \bigl\{ f \in X^{*}: \langle x, f\rangle= \|x\|^{2} = \|f \|^{2} \bigr\} ,\quad \forall x \in X. $$

A point $x \in D(A)$ is called a zero of *A* if $Ax = 0$. The set of zeros of *A* is denoted by $A^{-1}0$.

Suppose that the Lyapunov functional $\phi: X \times X \rightarrow (0,+\infty)$ is defined as follows:

$$\phi(x,y) = \|x\|^{2}-2 \bigl\langle x,j(y) \bigr\rangle + \|y \|^{2},\quad \forall x , y \in X, j(y) \in J(y). $$

Let *T* be a single-valued mapping of *K* into itself.

1. If $Tp = p$, then *p* is called a fixed point of *T*. And $\operatorname{Fix}(T)$ denotes the set of fixed points of *T*;
2. If there exists a sequence $\{x_{n}\}\subset K$ which converges weakly to $p\in K$ such that $x_{n} - Tx_{n} \rightarrow0$, as $n \rightarrow\infty$, then *p* is called an asymptotic fixed point of *T* [2]. And $\widehat{\operatorname{Fix}}(T)$ denotes the set of asymptotic fixed points of *T*;
3. If there exists a sequence $\{x_{n}\}\subset K$ which converges strongly to $p\in K$ such that $x_{n} - Tx_{n} \rightarrow0$, as $n \rightarrow\infty$, then *p* is called a strong asymptotic fixed point of *T* [2]. And $\widetilde{\operatorname{Fix}}(T)$ denotes the set of strong asymptotic fixed points of *T*;
4. *T* is called strongly relatively non-expansive [2] if $\widehat{\operatorname{Fix}}(T) = \operatorname{Fix}(T)\neq\emptyset$ and $\phi(p, Tx)\leq\phi (p,x)$ for $x \in K$ and $p \in \operatorname{Fix}(T)$;
5. *T* is called weakly relatively non-expansive [2] if $\widetilde{\operatorname{Fix}}(T) = \operatorname{Fix}(T)\neq\emptyset$ and $\phi(p, Tx)\leq\phi (p,x)$ for $x \in K$ and $p \in \operatorname{Fix}(T)$.

If *X* is a real reflexive and strictly convex Banach space and *K* is a non-empty closed and convex subset of *X*, then for each $x \in X$ there exists a unique point $x_{0} \in K$ such that $\|x - x_{0}\| = \inf \{ \|x - y\|: y \in K\}$. In this case, we can define the metric projection mapping $P_{K}: X \rightarrow K$ by $P_{K}x = x_{0}$ for $\forall x \in X$ [3].

If *X* is a real reflexive, strictly convex, and smooth Banach space and *K* is a non-empty closed and convex subset of *X*, then for $\forall x \in X$, there exists a unique point $x_{0} \in K$ such that $\phi(x_{0}, x) = \inf \{\phi(y,x) : y \in K\}$. In this case, we can define the generalized projection mapping $\Pi_{K}: X \rightarrow K$ by $\Pi_{K} x = x_{0}$ for $\forall x \in X$ [3].

Note that if *X* is a Hilbert space *H*, then $P_{K}$ and $\Pi_{K}$ are coincidental.

Since maximal monotone operators and weakly (or strongly) relatively non-expansive mappings have close connection with practical problems, one has a good reason to study them. During past years, much work has been done in designing iterative schemes to approximate a common element of the set of zeros of maximal monotone operators and the set of fixed points of weakly (or strongly) relatively non-expansive mappings. Among them, a projection iterative scheme is considered as one of the effective iterative schemes which almost always generates strongly convergent iterative sequences (see [4–8] and the references therein). Next, we list some recent closely related work.

Klin-eam et al. [5] presented the following projection iterative scheme for maximal monotone operator *A* and two strongly relatively non-expansive mappings *B* and *C* in a real uniformly convex and uniformly smooth Banach space *X*.

1.1 $$ \textstyle\begin{cases} v_{n} = J^{-1}[\beta_{n} Jx_{n}+(1-\beta_{n})JC(J+r_{n}A)^{-1}Jx_{n}], \\ y_{n} = J^{-1}[\alpha_{n} Jx_{n}+(1-\alpha_{n})JBv_{n}], \\ H_{n}= \{p \in K: \phi(p,y_{n})\leq\phi(p,x_{n})\}, \\ V_{n} = \{p \in K: \langle p - x_{n}, Jx_{1} - Jx_{n}\rangle\leq0\}, \\ x_{n+1}= \Pi_{H_{n} \cap V_{n}}(x_{1}),\quad n \in N. \end{cases} $$

Then $\{x_{n}\}$ generated by (1.1) converges strongly to $\Pi_{A^{-1}0 \cap \operatorname{Fix}(B)\cap \operatorname{Fix}(C)}(x_{1})$.

Compared to (1.1), the following so-called monotone projection iterative scheme for maximal monotone operator *A* and strongly relatively non-expansive mapping *B* in a real uniformly convex and uniformly smooth Banach space *X* is presented [4].

1.2 $$ \textstyle\begin{cases} x_{1} \in X,\qquad r_{1} > 0, \\ y_{n} = (J+r_{n}A)^{-1}J(x_{n}+e_{n}), \\ z_{n} = J^{-1}[\alpha_{n} Jx_{n}+(1-\alpha_{n})Jy_{n}], \\ u_{n} = J^{-1}[\beta_{n} Jx_{n}+(1-\beta_{n})JBz_{n}], \\ H_{1}= \{p \in X: \phi(p,z_{1})\leq\alpha_{1}\phi(p,x_{1})+(1-\alpha_{1})\phi (p,x_{1}+e_{1})\}, \\ V_{1} = \{p \in X: \phi(p,u_{1})\leq\beta_{1}\phi(p,x_{1})+(1-\beta_{1})\phi (p,z_{1})\}, \\ W_{1} = X, \\ H_{n}= \{p \in H_{n-1}\cap V_{n-1}\cap W_{n-1}: \phi(p,z_{n})\leq \alpha_{n}\phi(p,x_{n})+(1-\alpha_{n})\phi(p,x_{n}+e_{n})\}, \\ V_{n} = \{p \in H_{n-1}\cap V_{n-1}\cap W_{n-1}: \phi(p,u_{n})\leq \beta_{n}\phi(p,x_{n})+(1-\beta_{n})\phi(p,z_{n})\}, \\ W_{n} = \{p \in H_{n-1}\cap V_{n-1}\cap W_{n-1}: \langle p - x_{n}, Jx_{1} - Jx_{n}\rangle\leq0\}, \\ x_{n+1}= \Pi_{H_{n} \cap V_{n} \cap W_{n}}(x_{1}), \quad n \in N. \end{cases} $$

Then $\{x_{n}\}$ generated by (1.2) converges strongly to $\Pi_{A^{-1}0 \cap \operatorname{Fix}(B)}(x_{1})$.

In recent work, Wei et al. [8] extended the corresponding topic to the case for infinite maximal monotone operators $A_{i}$ and infinite weakly relatively non-expansive mappings $B_{i}$.

1.3 $$ \textstyle\begin{cases} x_{1} \in X,\qquad r_{1,i}\in(0,+\infty),\quad i \in N, \\ y_{n,i} = (J+r_{n,i}A_{i})^{-1}J(x_{n}+e_{n}),\quad i \in N, \\ z_{n,i} = J^{-1}[\alpha_{n}Jx_{n}+(1-\alpha_{n})JB_{i}y_{n,i}], \quad i \in N, \\ V_{1} = X = W_{1}, \\ V_{n+1,i} = \{p \in X: \langle y_{n,i}-p, J(x_{n}+e_{n}) - Jy_{n,i}\rangle \geq0\}, \quad i \in N, \\ V_{n+1}= (\bigcap_{i = 1}^{\infty} V_{n+1,i})\cap V_{n}, \\ W_{n+1,i} = \{p \in V_{n+1,i}: \phi(p,z_{n,i}) \leq\alpha_{n} \phi (p,x_{n}) + (1-\alpha_{n})\phi(p,y_{n,i})\},\quad i \in N, \\ W_{n+1}= (\bigcap_{i = 1}^{\infty} W_{n+1,i})\cap W_{n}, \\ U_{n+1} = \{p \in W_{n+1}: \|x_{1} - p\|^{2} \leq\|P_{W_{n+1}}(x_{1})- x_{1}\| ^{2}+\lambda_{n+1}\}, \\ x_{n+1} \in U_{n+1},\quad n \in N. \end{cases} $$

Then $\{x_{n}\}$ generated by (1.3) converges strongly to

$$P_{\bigcap _{n=1}^{\infty}W_{n}}(x_{1})\in\Biggl(\bigcap _{i = 1}^{\infty }A_{i}^{-1}0\Biggr)\cap \Biggl(\bigcap_{i = 1}^{\infty}\operatorname{Fix}(B_{i}) \Biggr). $$

Compared to traditional (monotone) projection iterative schemes (e.g., (1.1) and (1.2)), some different ideas in (1.3) can be seen. (1) Metric projection mapping $P_{W_{n+1}}$ instead of generalized projection mapping Π is involved in (1.3). (2) The iterative item $x_{n+1}$ can be chosen arbitrarily in the set $U_{n+1}$, while $x_{n+1}$ in both (1.1) and (1.2) and some others are needed to be the unique value of generalized projection mapping Π. (3) $\{x_{n}\}$ in (1.3) converges strongly to the unique value of metric projection mapping *P*, while $\{ x_{n}\}$ in both (1.1) and (1.2) converges strongly to the unique value of the generalized projection mapping Π.

A special case of (1.3) is presented as Corollary 2.13 in [8]. Now, we rewrite it as follows:

1.4 $$ \textstyle\begin{cases} x_{1} \in H, \qquad e_{1} \in H, \\ y_{n} = (I+r_{n}A)^{-1}(x_{n}+e_{n}), \\ z_{n} = \alpha_{n}x_{n}+(1-\alpha_{n})By_{n}, \\ U_{1} = H = V_{1}, \\ U_{n+1} = \{p \in U_{n}: (y_{n}-p)(x_{n}+e_{n} - y_{n}) \geq0, \\ \|p-z_{n}\|^{2} \leq\alpha_{n}\|p-x_{n}\|^{2} + (1-\alpha_{n})\|p-y_{n}\|^{2}\} , \\ V_{n+1} = \{p \in U_{n+1}: \|x_{1} - p\|^{2} \leq\|P_{U_{n+1}}(x_{1})- x_{1}\| ^{2}+\lambda_{n+1}\}, \\ x_{n+1} \in V_{n+1},\quad n \in N. \end{cases} $$

Based on iterative scheme (1.4), an iterative sequence is defined as follows after taking $H = (-\infty,+\infty)$, $Ax = 2x$, $Bx = x$ for $x \in(-\infty,+\infty)$, $e_{n} = \alpha_{n} = \lambda_{n} = \frac{1}{n}$, and $r_{n} = 2^{n-1}$:

1.5 $$ \textstyle\begin{cases} x_{1} = 1, \\ y_{n} = \frac{x_{n}+e_{n}}{1+2r_{n}},\quad n \in N, \\ x_{n+1}= \frac{x_{1}+y_{n}-\sqrt{(x_{1}-y_{n})^{2}+\lambda_{n+1}}}{2},\quad n \in N. \end{cases} $$

A computational experiment based on (1.5) is conducted in [8], from which we can see the effectiveness of iterative scheme (1.4).

Inspired by the work of [8], three questions come to our mind. (1) In iterative scheme (1.3), in each iterative step *n*, countable sets $V_{n+1,i}$ and $W_{n+1,i}$ are needed to be evaluated. It is formidable. Can we avoid it? (2) $x_{n+1}$ in either (1.3) or (1.4) can be chosen arbitrarily in a set, can a different choice of $x_{n+1}$ in $V_{n+1}$ lead to a different rate of convergence? (3) Which one is better, our new one or those in [8]? In this paper, we shall answer the questions, construct new simple projection sets in theoretical sense, and do computational experiments for some special cases.

## Preliminaries

In this section, we list some definitions and results we need later. The modulus of convexity of *X*, $\delta_{X}: [0,2] \rightarrow[0,1]$, is defined as follows [9]:

$$\delta_{X}(\epsilon) = \sup \biggl\{ 1-\frac{\|x+y\|}{2}: x,y \in X, \|x\|= \| y\| = 1, \|x - y\| \geq \epsilon \biggr\} $$

for $\forall\epsilon\in[0,2]$. A Banach space *X* is called uniformly convex [9] if $\delta_{X}(\epsilon)> 0$ for $\forall \epsilon\in[0,2]$. A Banach space *X* is called uniformly smooth [9] if the limit $\lim_{t \rightarrow0}\frac{\|x+ty\|-\|x\|}{t}$ is attained uniformly for $(x,y)\in X\times X$ with $\|x\|= \|y\| = 1$.

*X* is said to have Property (H): if for every sequence $\{x_{n}\} \subset X$ converging weakly to $x \in X$ and $\|x_{n}\| \rightarrow\|x\| $, one has $x_{n} \rightarrow x$, as $n \rightarrow\infty$. The uniformly convex and uniformly smooth Banach space *X* has Property (H).

It is well known that if *X* is a real uniformly convex and uniformly smooth Banach space, then the normalized duality mapping *J* is single-valued, surjective and $J(kx) = kJ(x)$ for $x \in X$ and $k \in (-\infty,+\infty)$. Moreover, $J^{-1}$ is also the normalized duality mapping from $X^{*}$ into *X*, and both *J* and $J^{-1}$ are uniformly continuous on each bounded subset of *X* or $X^{*}$, respectively [9].

### Lemma 2.1

([2])

*Suppose that* *X* *is a uniformly convex and uniformly smooth Banach space and* *K* *is a non*-*empty closed and convex subset of* *X*. *If* $B: K \rightarrow K$ *is weakly relatively non*-*expansive*, *then* $\operatorname{Fix}(B)$ *is a closed and convex subset of* *X*.

### Lemma 2.2

([1])

*Let* $A : X \rightarrow2^{X^{*}}$ *be a maximal monotone operator*, *then*

1. $A^{-1}0$ *is a closed and convex subset of* *X*;
2. *if* $x_{n} \rightarrow x$ *and* $y_{n} \in Ax_{n}$ *with* $y_{n} \rightharpoonup y$, *or* $x_{n} \rightharpoonup x$ *and* $y_{n} \in Ax_{n}$ *with* $y_{n} \rightarrow y$, *then* $x \in D(A)$ *and* $y \in Ax$.

### Lemma 2.3

([8])

*Let* *K* *be a non*-*empty closed and convex subset of a uniformly smooth Banach space* *X*. *Let* $x \in X$ *and* $x_{0} \in K$. *Then* $\phi(x_{0}, x) = \inf_{y \in K} \phi(y,x)$ *if and only if* $\langle x_{0} - z, Jx - Jx_{0}\rangle\geq0$ *for all* $z \in K$.

### Lemma 2.4

([10])

*Let* *X* *be a real uniformly smooth and uniformly convex Banach space*, *and let* $\{x_{n}\}$ *and* $\{y_{n}\}$ *be two sequences of* *X*. *If either* $\{x_{n}\}$ *or* $\{y_{n}\}$ *is bounded and* $\phi (x_{n},y_{n}) \rightarrow0$ *as* $n \rightarrow\infty$, *then* $x_{n} - y_{n} \rightarrow0$ *as* $n \rightarrow\infty$.

### Lemma 2.5

([11])

*Let* *X* *be a real uniformly smooth and uniformly convex Banach space and* $A: X \rightarrow2^{X^{*}}$ *be a maximal monotone operator with* $A^{-1}0 \neq\emptyset$. *Then*, *for* $\forall x \in X$, $\forall y \in A^{-1}0$, *and* $r > 0$, *one has* $\phi(y, (J+rA)^{-1}Jx)+ \phi((J+rA)^{-1}Jx, x) \leq\phi(y,x)$.

Let $\{K_{n}\}$ be a sequence of non-empty closed and convex subsets of *X*. Then the strong lower limit of $\{K_{n}\}$, $s\mbox{-}\liminf K_{n}$, is defined as the set of all $x \in X$ such that there exists $x_{n} \in K_{n}$ for almost all *n* and it tends to *x* as $n \rightarrow\infty$ in the norm; the weak upper limit of $\{K_{n}\}$, $w\mbox{-}\limsup K_{n}$, is defined as the set of all $x \in X$ such that there exists a subsequence $\{K_{n_{m}}\}$ of $\{K_{n}\}$ and $x_{n_{m}} \in K_{n_{m}}$ for every $n_{m}$ and it tends to *x* as $n_{m} \rightarrow\infty$ in the weak topology; the limit of $\{K_{n}\}$, $\lim K_{n}$, is the common value when $s\mbox{-}\liminf K_{n} = w\mbox{-}\limsup K_{n}$ [12].

### Lemma 2.6

([12])

*Let* $\{K_{n}\}$ *be a decreasing sequence of closed and convex subsets of* *X*, *i*.*e*., $K_{n} \subset K_{m}$ *if* $n \geq m$. *Then* $\{K_{n}\}$ *converges in* *X* *and* $\lim K_{n} = \bigcap_{n = 1}^{\infty} K_{n}$.

### Lemma 2.7

([13])

*Suppose that* *X* *is a real uniformly convex Banach space*. *If* $\lim K_{n}$ *exists and is not empty*, *then* $\{P_{K_{n}}x\}$ *converges weakly to* $P_{\lim K_{n}}x$ *for every* $x \in X$. *Moreover*, *if* *X* *has Property* (H), *the convergence is in norm*.

### Lemma 2.8

([14])

*Let* *X* *be a real uniformly convex Banach space and* $r \in(0,+\infty)$. *Then there exists a continuous*, *strictly increasing*, *and convex function* $\eta: [0, 2r] \rightarrow[0, +\infty )$ *with* $\eta(0) = 0$ *such that* $\|kx+(1-k)y\|^{2} \leq k\|x\|^{2} +(1-k)\| y\|^{2} - k(1-k)\eta(\|x - y\|)$ *for* $k \in[0,1], x,y \in X$ *with* $\|x\| \leq r$ *and* $\|y\| \leq r$.

### Lemma 2.9

([15])

*Let* *X* *be the same as that in Lemma *2.8. *Then there exists a continuous*, *strictly increasing*, *and convex function* $\eta: [0, 2r] \rightarrow[0, +\infty)$ *with* $\eta(0) = 0$ *such that* $\|\sum_{i = 1}^{\infty} k_{i}x_{i}\|^{2} \leq\sum_{i = 1}^{\infty} k_{i}\|x_{i}\|^{2} - k_{1}k_{m}\eta(\|x_{1} - x_{m}\|)$ *for all* $\{x_{n}\}_{n = 1}^{\infty}\subset\{x \in X: \|x\| \leq r\}$, $\{k_{n}\}_{n = 1}^{\infty}\subset(0,1)$ *with* $\sum_{n = 1}^{\infty} k_{n} = 1$ *and* $m \in N$.

## Main results

In this section, our discussion is based on the following conditions:

($I_{1}$)
*X* is a real uniformly convex and uniformly smooth Banach space and $J: X \rightarrow X^{*}$ is the normalized duality mapping;
($I_{2}$)
$A_{i}: X \rightarrow X^{*}$ is maximal monotone and $B_{i} : X \rightarrow X$ is weakly relatively non-expansive for each $i\in N$. And $(\bigcap_{i = 1}^{\infty}A_{i}^{-1}0)\cap(\bigcap_{i = 1}^{\infty}\operatorname{Fix}(B_{i})) \neq\emptyset$;
($I_{3}$)
$\{e_{n}\} \subset X$ is the error sequence such that $e_{n} \rightarrow0$, as $n \rightarrow\infty$;
($I_{4}$)
$\{r_{n,i}\}$ and $\{\lambda_{n}\}$ are two real number sequences in $(0,+\infty)$ with $\inf_{n}r_{n,i} > 0$ for $i \in N$ and $\lambda _{n} \rightarrow0$, as $n \rightarrow\infty$;
($I_{5}$)
$\{a_{n,i}\}$ and $\{b_{i}\}$ are two real number sequences in $(0,1)$ and $\sum_{i = 1}^{\infty} a_{n,i} = 1 = \sum_{i = 1}^{\infty} b_{i}$ for $n \in N$;
($I_{6}$)
$\{\alpha_{n}\}$ and $\{\beta_{n}\}$ are two real number sequences in $[0,1)$.

### Theorem 3.1

*Let* $\{x_{n}\}$ *be generated by the following iterative scheme*:

3.1 $$ \textstyle\begin{cases} x_{1} \in X, \qquad e_{1} \in X, \\ y_{n} = J^{-1}[\alpha_{n}Jx_{n}+(1-\alpha_{n})\sum_{i = 1}^{\infty} a_{n,i}J(J+r_{n,i}A_{i})^{-1}J(x_{n}+e_{n})], \\ z_{n} = J^{-1}[\beta_{n}Jx_{n}+(1-\beta_{n})\sum_{i = 1}^{\infty} b_{i}JB_{i}y_{n}], \\ U_{1} = X = V_{1}, \\ U_{n+1} = \{v \in U_{n}: \phi(v,y_{n}) \leq\alpha_{n} \phi(v,x_{n}) + (1-\alpha_{n})\phi(v,x_{n}+e_{n}), \\ \hphantom{U_{n+1} ={}}\phi(v,z_{n}) \leq\beta_{n} \phi(v,x_{n}) + (1-\beta_{n})\phi(v,y_{n})\}, \\ V_{n+1} = \{v \in U_{n+1}: \|x_{1} - v\|^{2} \leq\|P_{U_{n+1}}(x_{1})- x_{1}\| ^{2}+\lambda_{n+1}\}, \\ x_{n+1} \in V_{n+1}, \quad n \in N. \end{cases} $$

*If* $0 \leq \sup_{n}\alpha_{n} < 1$ *and* $0 \leq \sup_{n}\beta_{n} < 1$, *then* $x_{n} \rightarrow P_{\bigcap_{m = 1}^{\infty}U_{m}}(x_{1}) \in(\bigcap_{i = 1}^{\infty}A_{i}^{-1}0)\cap (\bigcap_{i = 1}^{\infty }\operatorname{Fix}(B_{i}))$, *as* $n \rightarrow\infty$.

### Proof

We split the proof into seven steps.

*Step* 1. $U_{n}$ is a non-empty closed and convex subset of *X* for each $n \in N$.

Noticing the definition of Lyapunov functional, we have

$$\begin{aligned}& \phi(v, y_{n}) \leq\alpha_{n} \phi(v, x_{n})+ (1-\alpha_{n}) \phi(v, x_{n}+e_{n}) \\& \quad \Longleftrightarrow\quad 2\alpha_{n} \langle v, Jx_{n}\rangle+2(1-\alpha _{n}) \bigl\langle v, J(x_{n}+e_{n}) \bigr\rangle - 2 \langle v, Jy_{n} \rangle \\& \hphantom{\quad \Longleftrightarrow\quad}\quad \leq \alpha_{n}\|x_{n} \|^{2}+ (1-\alpha_{n}) \|x_{n}+e_{n} \|^{2}- \|y_{n}\|^{2} \end{aligned}$$

and

$$\begin{aligned}& \phi(v, z_{n}) \leq\beta_{n} \phi(v, x_{n})+ (1-\beta_{n}) \phi(v, y_{n}) \\& \quad \Longleftrightarrow\quad 2\beta_{n} \langle v, Jx_{n}\rangle+2(1-\beta _{n})\langle v, Jy_{n} \rangle- 2 \langle v, Jz_{n}\rangle \\& \hphantom{\quad \Longleftrightarrow\quad}\quad \leq \beta_{n}\|x_{n} \|^{2}+ (1-\beta_{n}) \|y_{n}\|^{2}- \|z_{n}\|^{2}. \end{aligned}$$

Thus $U_{n}$ is closed and convex for each $n \in N$.

Next, we shall prove that $(\bigcap_{i = 1}^{\infty}A_{i}^{-1}0)\cap (\bigcap_{i = 1}^{\infty}\operatorname{Fix}(B_{i}))\subset U_{n}$, which implies that $U_{n} \neq\emptyset$.

For this, we shall use inductive method. Now, $\forall q \in(\bigcap_{i = 1}^{\infty}A_{i}^{-1}0)\cap(\bigcap_{i = 1}^{\infty }\operatorname{Fix}(B_{i}))$.

If $n=1$, then $q \in U_{1} = X$ is obviously true. In view of the convexity of $\|\cdot\|^{2}$ and Lemma 2.5, we have

$$\begin{aligned} \phi(q, y_{1}) =& \Vert q \Vert ^{2} - 2 \Biggl\langle q, \alpha _{1}Jx_{1}+(1-\alpha_{1})\sum _{i = 1}^{\infty }a_{1,i}J(J+r_{1,i}A_{i})^{-1}J(x_{1}+e_{1}) \Biggr\rangle \\ &{}+ \Biggl\Vert \alpha_{1}Jx_{1}+(1- \alpha_{1})\sum_{i = 1}^{\infty }a_{1,i}J(J+r_{1,i}A_{i})^{-1}J(x_{1}+e_{1}) \Biggr\Vert ^{2} \\ \leq& \Vert q \Vert ^{2} - 2 \alpha_{1}\langle q, Jx_{1}\rangle-2(1-\alpha_{1})\sum _{i = 1}^{\infty}a_{1,i} \bigl\langle q, J(J+r_{1,i}A_{i})^{-1}J(x_{1}+e_{1}) \bigr\rangle \\ &{}+ \alpha_{1} \Vert x_{1} \Vert ^{2}+(1- \alpha_{1})\sum_{i = 1}^{\infty}a_{1,i} \bigl\Vert (J+r_{1,i}A_{i})^{-1}J(x_{1}+e_{1}) \bigr\Vert ^{2} \\ =& \alpha_{1} \phi(q, x_{1}) + (1-\alpha_{1}) \sum_{i = 1}^{\infty}a_{1,i}\phi \bigl(q, (J+r_{1,i}A_{i})^{-1}J(x_{1}+e_{1}) \bigr) \\ \leq&\alpha_{1} \phi(q, x_{1}) + (1-\alpha_{1}) \phi(q, x_{1}+e_{1}). \end{aligned}$$

Moreover, from the definition of weakly relatively non-expansive mapping, we have

$$\begin{aligned} \phi(q, z_{1}) \leq& \|q\|^{2} - 2 \beta_{1} \langle q, Jx_{1}\rangle-2(1-\beta_{1})\sum _{i = 1}^{\infty}b_{i}\langle q, JB_{i}y_{1}\rangle \\ &{}+ \beta_{1}\|x_{1}\|^{2}+(1- \beta_{1})\sum_{i = 1}^{\infty}b_{i} \|B_{i}y_{1}\|^{2} \\ =& \beta_{1} \phi(q, x_{1}) + (1-\beta_{1}) \sum_{i = 1}^{\infty}b_{i}\phi (q,B_{i}y_{1})\leq\beta_{1} \phi(q, x_{1}) + (1-\beta_{1})\phi(q, y_{1}). \end{aligned}$$

Thus $q \in U_{2}$.

Suppose the result is true for $n = k+1$. Then, if $n = k+2$, we have

$$\begin{aligned} \phi(q, y_{k+1}) =& \Vert q \Vert ^{2} - 2 \Biggl\langle q, \alpha _{k+1}Jx_{k+1}+(1-\alpha_{k+1})\sum _{i = 1}^{\infty }a_{k+1,i}J(J+r_{k+1,i}A_{i})^{-1}J(x_{k+1}+e_{k+1}) \Biggr\rangle \\ &{}+ \Biggl\Vert \alpha_{k+1}Jx_{k+1}+(1- \alpha_{k+1})\sum_{i = 1}^{\infty }a_{k+1,i}J(J+r_{k+1,i}A_{i})^{-1}J(x_{k+1}+e_{k+1}) \Biggr\Vert ^{2} \\ \leq& \Vert q \Vert ^{2}- 2 \alpha_{k+1}\langle q, Jx_{k+1}\rangle \\ &{}-2(1-\alpha _{k+1})\sum _{i = 1}^{\infty}a_{k+1,i} \bigl\langle q, J(J+r_{k+1,i}A_{i})^{-1}J(x_{k+1}+e_{k+1}) \bigr\rangle \\ &{}+ \alpha_{k+1} \Vert x_{k+1} \Vert ^{2}+(1- \alpha_{k+1})\sum_{i = 1}^{\infty }a_{k+1,i} \bigl\Vert (J+r_{k+1,i}A_{i})^{-1}J(x_{k+1}+e_{k+1}) \bigr\Vert ^{2} \\ =& \alpha_{k+1} \phi(q, x_{k+1}) + (1-\alpha_{k+1}) \sum_{i = 1}^{\infty }a_{k+1,i}\phi \bigl(q, (J+r_{k+1,i}A_{i})^{-1}J(x_{k+1}+e_{k+1}) \bigr) \\ \leq&\alpha_{k+1} \phi(q, x_{k+1}) + (1-\alpha_{k+1}) \phi(q, x_{k+1}+e_{k+1}). \end{aligned}$$

Moreover,

$$\begin{aligned} \phi(q, z_{k+1}) \leq& \Vert q \Vert ^{2} - 2 \beta_{k+1}\langle q, Jx_{k+1}\rangle-2(1-\beta_{k+1}) \sum_{i = 1}^{\infty}b_{i}\langle q, JB_{i}y_{k+1}\rangle \\ &{}+ \beta_{k+1} \Vert x_{k+1} \Vert ^{2}+(1- \beta_{k+1})\sum_{i = 1}^{\infty}b_{i} \Vert B_{i}y_{k+1} \Vert ^{2} \\ =& \beta_{k+1} \phi(q, x_{k+1}) + (1-\beta_{k+1}) \sum_{i = 1}^{\infty }b_{i} \phi(q,B_{i}y_{k+1}) \\ \leq&\beta_{k+1} \phi(q, x_{k+1}) + (1-\beta_{k+1}) \phi(q, y_{k+1}). \end{aligned}$$

Then $q \in U_{k+2}$. Therefore, by induction, $(\bigcap_{i = 1}^{\infty }A_{i}^{-1}0)\cap(\bigcap_{i = 1}^{\infty}\operatorname{Fix}(B_{i}))\subset U_{n}$ for $n \in N$.

*Step* 2. $P_{U_{n+1}}(x_{1}) \rightarrow P_{\bigcap_{m = 1}^{\infty }U_{m}}(x_{1})$, as $n \rightarrow\infty$.

It follows from Lemma 2.6 that $\lim U_{n}$ exists and $\lim U_{n} = \bigcap_{n = 1}^{\infty} U_{n} \neq\emptyset$. Since *X* has Property (H), then Lemma 2.7 implies that $P_{U_{n+1}}(x_{1}) \rightarrow P_{\bigcap_{m = 1}^{\infty}U_{m}}(x_{1})$, as $n \rightarrow\infty$.

*Step* 3. $V_{n} \neq\emptyset$, for $N \cup\{0\}$, which ensures that $\{x_{n}\}$ is well defined.

Since $\|P_{U_{n+1}}(x_{1}) - x_{1}\| = \inf_{y \in U_{n+1}}\|y - x_{1}\|$, then for $\lambda_{n+1}$, there exists $\delta_{n+1} \in U_{n+1}$ such that $\|x_{1} - \delta_{n+1}\|^{2} \leq(\inf_{y \in U_{n+1}} \|x_{1} - y\|)^{2} + \lambda_{n+1} = \|P_{U_{n+1}}(x_{1})-x_{1}\|^{2}+ \lambda_{n+1}$. This ensures that $V_{n+1}\neq\emptyset$ for $n \in N \cup\{0\}$.

*Step* 4. Both $\{x_{n}\}$ and $\{P_{U_{n+1}}(x_{1})\}$ are bounded.

Since $\lambda_{n} \rightarrow0$, then there exists $M_{1} > 0$ such that $\lambda_{n} < M_{1}$ for $n \in N$. Step 2 implies that $\{ P_{U_{n+1}}(x_{1})\}$ is bounded, and then there exists $M_{2} > 0$ such that $\|P_{U_{n+1}}(x_{1})\| \leq M_{2}$ for $n \in N$. Set $M = (M_{2}+\| x_{1}\|)^{2}+M_{1}$. Since $x_{n+1} \in V_{n+1}$, then $\|x_{1} - x_{n+1}\|^{2} \leq\|P_{U_{n+1}}(x_{1})-x_{1}\|^{2}+ \lambda _{n+1}\leq M$, $\forall n \in N$. Thus $\{x_{n}\}$ is bounded.

*Step* 5. $x_{n+1} - P_{U_{n+1}}(x_{1})\rightarrow0$, as $n \rightarrow \infty$.

Since $x_{n+1} \in V_{n+1}\subset U_{n+1}$ and $U_{n}$ is a convex subset of *X*, then for $\forall k \in(0,1)$, $kP_{U_{n+1}}(x_{1})+(1-k)x_{n+1}\in U_{n+1}$. Thus

3.2 $$ \bigl\Vert P_{U_{n+1}}(x_{1}) - x_{1} \bigr\Vert \leq \bigl\Vert k P_{U_{n+1}}(x_{1})+(1-k)x_{n+1}-x_{1} \bigr\Vert . $$

Since $\{x_{n}\}$ is bounded, it follows from (3.2) and Lemma 2.8 that

$$\begin{aligned} \bigl\Vert P_{U_{n+1}}(x_{1}) - x_{1} \bigr\Vert ^{2} \leq& \bigl\Vert k P_{U_{n+1}}(x_{1})+(1-k)x_{n+1}-x_{1} \bigr\Vert ^{2} \\ \leq& k \bigl\Vert P_{U_{n+1}}(x_{1})- x_{1} \bigr\Vert ^{2} + (1-k) \Vert x_{n+1}-x_{1} \Vert ^{2} \\ &{}-k(1-k)\eta \bigl( \bigl\Vert P_{U_{n+1}}(x_{1})-x_{n+1} \bigr\Vert \bigr). \end{aligned}$$

Therefore, $k\eta(\|P_{U_{n+1}}(x_{1})-x_{n+1}\|)\leq\|x_{n+1}-x_{1}\|^{2}-\| P_{U_{n+1}}(x_{1})-x_{1}\|^{2} \leq\lambda_{n+1}$. Letting $k \rightarrow1$ first and then $n \rightarrow\infty$, we know that $P_{U_{n+1}}(x_{1})-x_{n+1} \rightarrow0$, as $n \rightarrow\infty$.

*Step* 6. $x_{n} \rightarrow P_{\bigcap_{m = 1}^{\infty}U_{m}}(x_{1})$, $y_{n} \rightarrow P_{\bigcap_{m = 1}^{\infty}U_{m}}(x_{1})$ and $z_{n} \rightarrow P_{\bigcap_{m = 1}^{\infty}U_{m}}(x_{1})$, as $n \rightarrow \infty$.

From Step 2 and Step 5, we know that $x_{n} \rightarrow P_{\bigcap_{m = 1}^{\infty}U_{m}}(x_{1})$, as $n \rightarrow\infty$. And then $x_{n+1} - x_{n} \rightarrow0$, as $n \rightarrow\infty$. Since $x_{n+1} \in V_{n+1}\subset U_{n+1}$ and $e_{n} \rightarrow0$, then

$$\begin{aligned} 0 \leq&\phi(x_{n+1}, y_{n})\leq\alpha_{n} \phi(x_{n+1}, x_{n})+ (1-\alpha_{n}) \phi(x_{n+1}, x_{n}+e_{n}) \\ =& \alpha_{n} \Vert x_{n+1} \Vert ^{2}+ \alpha_{n} \Vert x_{n} \Vert ^{2} - 2 \alpha_{n} \langle x_{n+1}, Jx_{n}\rangle \\ &{}+(1-\alpha_{n}) \Vert x_{n+1} \Vert ^{2} + (1-\alpha_{n}) \Vert x_{n}+e_{n} \Vert ^{2} -2 (1-\alpha _{n}) \bigl\langle x_{n+1}, J(x_{n}+e_{n}) \bigr\rangle \\ =& \Vert x_{n+1} \Vert ^{2} - \alpha_{n} \Vert x_{n} \Vert ^{2} - (1-\alpha_{n}) \Vert x_{n}+e_{n} \Vert ^{2} \\ &{}+2\alpha_{n}\langle x_{n}-x_{n+1}, Jx_{n}\rangle +2 (1-\alpha_{n}) \bigl\langle x_{n}+e_{n}-x_{n+1}, J(x_{n}+e_{n}) \bigr\rangle \\ \leq& \bigl( \Vert x_{n+1} \Vert ^{2}- \Vert x_{n}+e_{n} \Vert ^{2} \bigr)+ \alpha_{n} \bigl( \Vert x_{n}+e_{n} \Vert ^{2} - \Vert x_{n} \Vert ^{2} \bigr)+2 \alpha_{n} \Vert x_{n} \Vert \Vert x_{n+1}-x_{n} \Vert \\ &{}+2(1-\alpha_{n}) \Vert x_{n}+e_{n} \Vert \Vert x_{n}+e_{n}-x_{n+1} \Vert \rightarrow0. \end{aligned}$$

Then Lemma 2.4 implies that $x_{n+1} - y_{n}\rightarrow0$ and then $y_{n} \rightarrow P_{\bigcap_{m = 1}^{\infty}U_{m}}(x_{1})$, as $n \rightarrow \infty$.

Since $x_{n+1} \in V_{n+1}\subset U_{n+1}$ and *J* is uniformly continuous on each bounded subset of *X*, then

$$\begin{aligned} 0 \leq&\phi(x_{n+1}, z_{n})\leq\beta_{n} \phi(x_{n+1}, x_{n})+ (1-\beta_{n}) \phi(x_{n+1}, y_{n}) \\ =& \beta_{n} \bigl(\langle x_{n+1}, Jx_{n+1}- Jx_{n}\rangle+\langle x_{n} - x_{n+1}, Jx_{n}\rangle \bigr)+(1-\beta_{n})\phi(x_{n+1},y_{n}) \\ \leq&\beta_{n}\|x_{n+1}\|\|Jx_{n+1}- Jx_{n}\|+\beta_{n}\|x_{n}\|\|x_{n+1}-x_{n} \| +(1-\beta_{n})\phi(x_{n+1},y_{n})\rightarrow0. \end{aligned}$$

Using Lemma 2.4 again, we have $x_{n+1} - z_{n}\rightarrow0$ and then $z_{n} \rightarrow P_{\bigcap_{m = 1}^{\infty}U_{m}}(x_{1})$, as $n \rightarrow\infty$.

*Step* 7. $P_{\bigcap_{m = 1}^{\infty}U_{m}}(x_{1}) \in(\bigcap_{i = 1}^{\infty}A_{i}^{-1}0) \cap(\bigcap_{i = 1}^{\infty}\operatorname{Fix}(B_{i}))$.

First, we shall show that $P_{\bigcap_{m = 1}^{\infty}U_{m}}(x_{1}) \in \bigcap_{i = 1}^{\infty}A_{i}^{-1}0$.

From (3.1) and Lemma 2.5, for $\forall q \in(\bigcap_{i = 1}^{\infty }A_{i}^{-1}0)\cap(\bigcap_{i = 1}^{\infty}\operatorname{Fix}(B_{i}))$, we have

$$\begin{aligned} \phi(q, y_{n}) \leq&\alpha_{n} \phi(q, x_{n})+ (1-\alpha _{n})\sum_{i = 1}^{\infty} a_{n,i}\phi \bigl(q, (J+r_{n,i}A_{i})^{-1}J(x_{n}+e_{n}) \bigr) \\ \leq&\alpha_{n} \phi(q, x_{n}) \\ &{}+ (1-\alpha_{n}) \sum_{i = 1}^{\infty} a_{n,i} \bigl[ \phi(q, x_{n}+e_{n})-\phi \bigl((J+r_{n,i}A_{i})^{-1}J(x_{n}+e_{n}),x_{n}+e_{n} \bigr) \bigr]. \end{aligned}$$

Then

$$\begin{aligned}& (1-\alpha_{n})\sum_{i = 1}^{\infty} a_{n,i}\phi \bigl((J+r_{n,i}A_{i})^{-1}J(x_{n}+e_{n}),x_{n}+e_{n} \bigr) \\& \quad \leq\alpha_{n} \phi(q, x_{n})-\phi(q, y_{n})+ (1-\alpha_{n})\phi(q, x_{n}+e_{n}) \\& \quad = \alpha_{n} \bigl[\phi(q, x_{n})- \phi(q, x_{n}+e_{n}) \bigr]+ \bigl[\phi(q, x_{n}+e_{n})- \phi(q, y_{n}) \bigr] \\& \quad \leq \Vert x_{n} \Vert ^{2} - \Vert x_{n}+e_{n} \Vert ^{2}+2 \Vert q \Vert \bigl\Vert J(x_{n}+e_{n})-Jx_{n} \bigr\Vert \\& \qquad {}+ \Vert x_{n}+e_{n} \Vert ^{2}- \Vert y_{n} \Vert ^{2}+2 \Vert q \Vert \bigl\Vert Jy_{n}-J(x_{n}+e_{n}) \bigr\Vert . \end{aligned}$$

Since $0 \leq \sup_{n}\alpha_{n} < 1$, then $\sum_{i = 1}^{\infty} a_{n,i}\phi((J+r_{n,i}A_{i})^{-1}J(x_{n}+e_{n}),x_{n}+e_{n}) \rightarrow0$, which implies from Lemma 2.4 that $(J+r_{n,i}A_{i})^{-1}J(x_{n}+e_{n})- (x_{n}+e_{n}) \rightarrow0$, as $n \rightarrow\infty$. Thus from Step 6, $(J+r_{n,i}A_{i})^{-1}J(x_{n}+e_{n}) \rightarrow P_{\bigcap_{m = 1}^{\infty }U_{m}}(x_{1})$, as $n \rightarrow\infty$.

Denote $u_{n,i} = (J+r_{n,i}A_{i})^{-1}J(x_{n}+e_{n})$, then $Ju_{n,i} + r_{n,i}A_{i}u_{n,i} = J(x_{n}+e_{n})$. Since $u_{n,i} \rightarrow P_{\bigcap _{m = 1}^{\infty}U_{m}}(x_{1})$, $x_{n} \rightarrow P_{\bigcap_{m = 1}^{\infty}U_{m}}(x_{1})$, $e_{n} \rightarrow0$, $\inf_{n}r_{n,i} > 0$ and *J* is uniformly continuous on each bounded subset of *X*, then $A_{i}u_{n,i} \rightarrow0$ for $i \in N$, as $n \rightarrow\infty$. Using Lemma 2.2, $P_{\bigcap_{m = 1}^{\infty}U_{m}}(x_{1}) \in\bigcap_{i = 1}^{\infty}A_{i}^{-1}0$.

Next, we shall show that $P_{\bigcap_{m = 1}^{\infty}U_{m}}(x_{1})\in \bigcap_{i = 1}^{\infty}\operatorname{Fix}(B_{i})$.

Since $z_{n} = J^{-1}[ \beta_{n}Jx_{n} + (1-\beta_{n})\sum_{i = 1}^{\infty }b_{i}JB_{i}y_{n}]$, then $Jz_{n} - Jx_{n} = (1-\beta_{n})(\sum_{i = 1}^{\infty }b_{i}JB_{i}y_{n} - Jx_{n})$. Since both *J* and $J^{-1}$ are uniformly continuous on each bounded subset of *X*, $z_{n} \rightarrow P_{\bigcap _{m = 1}^{\infty}U_{m}}(x_{1})$, $x_{n} \rightarrow P_{\bigcap_{m = 1}^{\infty }U_{m}}(x_{1})$ and $0 \leq \sup_{n} \beta_{n} < 1$, then $\sum_{i = 1}^{\infty }b_{i}JB_{i}y_{n} - Jx_{n} \rightarrow0$, which implies that $J^{-1}(\sum_{i = 1}^{\infty}b_{i}JB_{i}y_{n}) \rightarrow P_{\bigcap_{m = 1}^{\infty }U_{m}}(x_{1})$, as $n \rightarrow\infty$.

Employing Lemma 2.9, for $\forall q \in(\bigcap_{i = 1}^{\infty }A_{i}^{-1}0)\cap(\bigcap_{i = 1}^{\infty}\operatorname{Fix}(B_{i}))$, we have

3.3 $$\begin{aligned}& \phi \Biggl(q, J^{-1} \Biggl(\sum_{i = 1}^{\infty }b_{i}JB_{i}y_{n} \Biggr) \Biggr) \\& \quad = \Vert q \Vert ^{2} - 2 \Biggl\langle q, \sum _{i = 1}^{\infty}b_{i}JB_{i}y_{n} \Biggr\rangle + \Biggl\Vert \sum_{i = 1}^{\infty}b_{i}JB_{i}y_{n} \Biggr\Vert ^{2} \\& \quad \leq \Vert q \Vert ^{2} - 2 \sum _{i = 1}^{\infty}b_{i}\langle q, JB_{i}y_{n}\rangle +\sum_{i = 1}^{\infty}b_{i} \Vert B_{i}y_{n} \Vert ^{2}-b_{1}b_{k} \eta \bigl( \Vert JB_{1}y_{n} - JB_{k} y_{n} \Vert \bigr) \\& \quad = \sum_{i = 1}^{\infty}b_{i} \phi(q,B_{i}y_{n}) - b_{1}b_{k}\eta \bigl( \Vert JB_{1}y_{n} - JB_{k} y_{n} \Vert \bigr). \end{aligned}$$

Since $Jy_{n} \rightarrow JP_{\bigcap_{m = 1}^{\infty}U_{m}}(x_{1})$ and $\sum_{i = 1}^{\infty}b_{i}JB_{i}y_{n} \rightarrow JP_{\bigcap_{m = 1}^{\infty }U_{m}}(x_{1})$, then from the definition of weakly relatively non-expansive mapping and (3.3), we have

$$\begin{aligned}& b_{1}b_{k}\eta \bigl( \Vert JB_{1}y_{n} - JB_{k} y_{n} \Vert \bigr) \\& \quad \leq\sum_{i = 1}^{\infty}b_{i} \phi(q,B_{i}y_{n}) - \phi \Biggl(q,J^{-1} \Biggl( \sum_{i = 1}^{\infty}b_{i}JB_{i}y_{n} \Biggr) \Biggr) \\& \quad \leq\sum_{i = 1}^{\infty}b_{i} \phi(q,y_{n}) - \phi \Biggl(q,J^{-1} \Biggl(\sum _{i = 1}^{\infty}b_{i}JB_{i}y_{n} \Biggr) \Biggr) \\& \quad = \Vert y_{n} \Vert ^{2} - 2 \langle q, Jy_{n}\rangle+ 2\sum_{i = 1}^{\infty }b_{i} \langle q, JB_{i}y_{n}\rangle- \Biggl\Vert \sum _{i = 1}^{\infty }b_{i}JB_{i}y_{n} \Biggr\Vert ^{2}\rightarrow0, \end{aligned}$$

as $n \rightarrow\infty$. This ensures that $JB_{1}y_{n} - JB_{k}y_{n} \rightarrow0$ for $k \neq1$, as $n \rightarrow\infty$.

Since $y_{n} \rightarrow P_{\bigcap_{m = 1}^{\infty}U_{m}}(x_{1})$, then $\{ y_{n}\}$ is bounded. Since $(\|q\| - \|B_{i}y_{n}\|)^{2} \leq\phi(q, B_{i}y_{n}) \leq\phi(q, y_{n})\leq(\|q\|+\|y_{n}\|)^{2}$, then $\|B_{i}y_{n}\| \leq\|q\|$ or $\|B_{i}y_{n}\| \leq2\|q\|+\|y_{n}\|$, $i \in N$. Set $K = \sup\{\|y_{n}\|: n \in N\}+2\|q\|$, then $K < +\infty$.

Since $\sum_{i = 1}^{\infty}b_{i} = 1$, then for $\forall\varepsilon> 0$, there exists $m_{0} \in N$ such that $\sum_{i = m_{0}+1}^{\infty}b_{i} < \frac{\varepsilon}{4K}$.

Since $JB_{1}y_{n} - JB_{k}y_{n} \rightarrow0$, as $n \rightarrow\infty$, for $\forall k \in\{1,2,\ldots, m_{0}\}$, then we can choose $n_{0} \in N$ such that $\|JB_{1}y_{n} - JB_{k}y_{n}\|< \frac{\varepsilon}{2}$ for all $n \geq n_{0}$ and $k \in\{2,\ldots, m_{0}\}$. Then, if $n \geq n_{0}$,

$$\begin{aligned} \Biggl\Vert JB_{1}y_{n} - \sum _{i = 1}^{\infty}b_{i}JB_{i}y_{n} \Biggr\Vert \leq&\sum_{i = 2}^{m_{0}}b_{i} \Vert JB_{1}y_{n} - JB_{i}y_{n} \Vert + \sum_{i = m_{0}+1}^{\infty}b_{i} \Vert JB_{1}y_{n} - JB_{i}y_{n} \Vert \\ < & \Biggl(\sum_{i = 2}^{m_{0}}b_{i} \Biggr)\frac{\varepsilon}{2}+ \Biggl(\sum_{i = m_{0}+1}^{\infty}b_{i} \Biggr)2K < \frac{\varepsilon}{2}+\frac{\varepsilon}{2} = \varepsilon. \end{aligned}$$

This implies that $JB_{1}y_{n} - \sum_{i = 1}^{\infty}b_{i}JB_{i}y_{n} \rightarrow0$, and then $JB_{1}y_{n} \rightarrow JP_{\bigcap_{m = 1}^{\infty}U_{m}}(x_{1})$, as $n \rightarrow\infty$. Thus $B_{1}y_{n} \rightarrow P_{\bigcap_{m = 1}^{\infty}U_{m}}(x_{1})$, as $n \rightarrow \infty$. Lemma 2.1 implies that $P_{\bigcap_{m = 1}^{\infty }U_{m}}(x_{1})\in \operatorname{Fix}(B_{1})$.

Repeating the above process for showing $P_{\bigcap_{m = 1}^{\infty }U_{m}}(x_{1})\in \operatorname{Fix}(B_{1})$, we can also prove that $P_{\bigcap_{m = 1}^{\infty}U_{m}}(x_{1})\in \operatorname{Fix}(B_{k})$, $\forall k \in N$. Therefore, $P_{\bigcap_{m = 1}^{\infty}U_{m}}(x_{1})\in\bigcap_{i = 1}^{\infty}\operatorname{Fix}(B_{i})$.

This completes the proof. □

### Theorem 3.2

*Let* $\{x_{n}\}$ *be generated by the following iterative scheme*:

3.4 $$ \textstyle\begin{cases} x_{1} \in X,\qquad e_{1} \in X, \\ y_{n} = J^{-1}[\alpha_{n}Jx_{1}+(1-\alpha_{n})\sum_{i = 1}^{\infty} a_{n,i}J(J+r_{n,i}A_{i})^{-1}J(x_{n}+e_{n})], \\ z_{n} = J^{-1}[\beta_{n}Jx_{1}+(1-\beta_{n})\sum_{i = 1}^{\infty} b_{i}JB_{i}y_{n}], \\ U_{1} = X = V_{1}, \\ U_{n+1} = \{v \in U_{n}: \phi(v,y_{n}) \leq\alpha_{n} \phi(v,x_{1}) + (1-\alpha_{n})\phi(v,x_{n}+e_{n}), \\ \hphantom{U_{n+1} ={}}\phi(v,z_{n}) \leq\beta_{n} \phi(v,x_{1}) + (1-\beta_{n})\phi(v,y_{n})\}, \\ V_{n+1} = \{v \in U_{n+1}: \|x_{1} - v\|^{2} \leq\|P_{U_{n+1}}(x_{1})- x_{1}\| ^{2}+\lambda_{n+1}\}, \\ x_{n+1} \in V_{n+1}, \quad n \in N. \end{cases} $$

*If* $\alpha_{n} \rightarrow0$, $\beta_{n} \rightarrow0$, *then* $x_{n} \rightarrow P_{\bigcap_{m = 1}^{\infty}U_{m}}(x_{1}) \in(\bigcap_{i = 1}^{\infty}A_{i}^{-1}0)\cap(\bigcap_{i = 1}^{\infty}\operatorname{Fix}(B_{i}))$, *as* $n \rightarrow\infty$.

### Proof

Copy Steps 2, 3, 4, and 5 of Theorem 3.1, and do small changes in Steps 1, 6, and 7 in the following way.

*Step* 1. $U_{n}$ is a non-empty closed and convex subset of *X*.

We notice that

$$\begin{aligned}& \phi(v, y_{n}) \leq\alpha_{n} \phi(v, x_{1})+ (1-\alpha_{n}) \phi(v, x_{n}+e_{n}) \\& \quad \Longleftrightarrow\quad 2\alpha_{n} \langle v, Jx_{1}\rangle+2(1-\alpha _{n}) \bigl\langle v, J(x_{n}+e_{n}) \bigr\rangle - 2 \langle v, Jy_{n} \rangle \\& \hphantom{\quad \Longleftrightarrow\quad }\quad \leq \alpha_{n}\|x_{1} \|^{2}+ (1-\alpha_{n}) \|x_{n}+e_{n} \|^{2}- \|y_{n}\|^{2} \end{aligned}$$

and

$$\begin{aligned}& \phi(v, z_{n}) \leq\beta_{n} \phi(v, x_{1})+ (1-\beta_{n}) \phi(v, y_{n}) \\& \quad \Longleftrightarrow\quad 2\beta_{n} \langle v, Jx_{1}\rangle+2(1-\beta _{n})\langle v, Jy_{n} \rangle- 2 \langle v, Jz_{n}\rangle \\& \hphantom{\quad \Longleftrightarrow\quad }\quad \leq \beta_{n}\|x_{1} \|^{2}+ (1-\beta_{n}) \|y_{n}\|^{2}- \|z_{n}\|^{2}. \end{aligned}$$

Thus $U_{n}$ is closed and convex for $n \in N$.

Next, we shall prove that $(\bigcap_{i = 1}^{\infty}A_{i}^{-1}0)\cap (\bigcap_{i = 1}^{\infty}\operatorname{Fix}(B_{i}))\subset U_{n}$, which ensures that $U_{n} \neq\emptyset$.

For this, we shall use inductive method. Now, $\forall q \in(\bigcap_{i = 1}^{\infty}A_{i}^{-1}0)\cap(\bigcap_{i = 1}^{\infty }\operatorname{Fix}(B_{i}))$.

If $n=1$, $q \in U_{1} = X $ is obviously true. In view of the convexity of $\|\cdot\|^{2}$ and Lemma 2.5, we have

$$\begin{aligned} \phi(q, y_{1}) =& \Vert q \Vert ^{2} - 2 \Biggl\langle q, \alpha _{1}Jx_{1}+(1-\alpha_{1})\sum _{i = 1}^{\infty }a_{1,i}J(J+r_{1,i}A_{i})^{-1}J(x_{1}+e_{1}) \Biggr\rangle \\ &{}+ \Biggl\Vert \alpha_{1}Jx_{1}+(1- \alpha_{1})\sum_{i = 1}^{\infty }a_{1,i}J(J+r_{1,i}A_{i})^{-1}J(x_{1}+e_{1}) \Biggr\Vert ^{2} \\ \leq& \Vert q \Vert ^{2} - 2 \alpha_{1}\langle q, Jx_{1}\rangle-2(1-\alpha_{1})\sum _{i = 1}^{\infty}a_{1,i} \bigl\langle q, J(J+r_{1,i}A_{i})^{-1}J(x_{1}+e_{1}) \bigr\rangle \\ &{}+ \alpha_{1} \Vert x_{1} \Vert ^{2}+(1- \alpha_{1})\sum_{i = 1}^{\infty}a_{1,i} \bigl\Vert (J+r_{1,i}A_{i})^{-1}J(x_{1}+e_{1}) \bigr\Vert ^{2} \\ =& \alpha_{1} \phi(q, x_{1}) + (1-\alpha_{1}) \sum_{i = 1}^{\infty}a_{1,i}\phi \bigl(q, (J+r_{1,i}A_{i})^{-1}J(x_{1}+e_{1}) \bigr) \\ \leq&\alpha_{1} \phi(q, x_{1}) + (1-\alpha_{1}) \phi(q, x_{1}+e_{1}). \end{aligned}$$

Moreover, from the definition of weakly relatively non-expansive mapping, we have

$$\begin{aligned} \phi(q, z_{1}) \leq& \Vert q \Vert ^{2} - 2 \beta_{1}\langle q, Jx_{1}\rangle-2(1-\beta_{1}) \sum_{i = 1}^{\infty}b_{i}\langle q, JB_{i}y_{1}\rangle \\ &{}+ \beta_{1} \Vert x_{1} \Vert ^{2}+(1-\beta_{1})\sum _{i = 1}^{\infty}b_{i} \Vert B_{i}y_{1} \Vert ^{2} \\ =& \beta_{1} \phi(q, x_{1}) + (1-\beta_{1}) \sum_{i = 1}^{\infty}b_{i}\phi (q,B_{i}y_{1})\leq\beta_{1} \phi(q, x_{1}) + (1-\beta_{1})\phi(q, y_{1}). \end{aligned}$$

Thus $q \in U_{2}$.

Suppose the result is true for $n = k+1$. Then, if $n = k+2$, we have

$$\begin{aligned} \phi(q, y_{k+1}) =& \Vert q \Vert ^{2} - 2 \Biggl\langle q, \alpha _{k+1}Jx_{1}+(1-\alpha_{k+1})\sum _{i = 1}^{\infty }a_{k+1,i}J(J+r_{k+1,i}A_{i})^{-1}J(x_{k+1}+e_{k+1}) \Biggr\rangle \\ &{}+ \Biggl\Vert \alpha_{k+1}Jx_{1}+(1- \alpha_{k+1})\sum_{i = 1}^{\infty }a_{k+1,i}J(J+r_{k+1,i}A_{i})^{-1}J(x_{k+1}+e_{k+1}) \Biggr\Vert ^{2} \\ \leq& \Vert q \Vert ^{2} - 2 \alpha_{k+1}\langle q, Jx_{1}\rangle \\ &{}-2(1-\alpha _{k+1})\sum _{i = 1}^{\infty}a_{k+1,i} \bigl\langle q, J(J+r_{k+1,i}A_{i})^{-1}J(x_{k+1}+e_{k+1}) \bigr\rangle \\ &{}+ \alpha_{k+1} \Vert x_{1} \Vert ^{2}+(1- \alpha_{k+1})\sum_{i = 1}^{\infty }a_{k+1,i} \bigl\Vert (J+r_{k+1,i}A_{i})^{-1}J(x_{k+1}+e_{k+1}) \bigr\Vert ^{2} \\ =& \alpha_{k+1} \phi(q, x_{1}) + (1-\alpha_{k+1}) \sum_{i = 1}^{\infty }a_{k+1,i}\phi \bigl(q, (J+r_{k+1,i}A_{i})^{-1}J(x_{k+1}+e_{k+1}) \bigr) \\ \leq&\alpha_{k+1} \phi(q, x_{1}) + (1-\alpha_{k+1}) \phi(q, x_{k+1}+e_{k+1}). \end{aligned}$$

Moreover,

$$\begin{aligned} \phi(q, z_{k+1}) \leq& \Vert q \Vert ^{2} - 2 \beta_{k+1}\langle q, Jx_{1}\rangle-2(1-\beta_{k+1}) \sum_{i = 1}^{\infty}b_{i}\langle q, JB_{i}y_{k+1}\rangle \\ &{}+ \beta_{k+1} \Vert x_{1} \Vert ^{2}+(1- \beta_{k+1})\sum_{i = 1}^{\infty}b_{i} \Vert B_{i}y_{k+1} \Vert ^{2} \\ =& \beta_{k+1} \phi(q, x_{1}) + (1-\beta_{k+1}) \sum_{i = 1}^{\infty }b_{i} \phi(q,B_{i}y_{k+1}) \\ \leq&\beta_{k+1} \phi(q, x_{1}) + (1-\beta_{k+1}) \phi(q, y_{k+1}). \end{aligned}$$

Then $q \in U_{k+2}$. Therefore, by induction, $\emptyset\neq(\bigcap_{i = 1}^{\infty}A_{i}^{-1}0)\cap(\bigcap_{i = 1}^{\infty }\operatorname{Fix}(B_{i}))\subset U_{n}$, for $n \in N$.

*Step* 6. $x_{n} \rightarrow P_{\bigcap_{m = 1}^{\infty}U_{m}}(x_{1})$, $y_{n} \rightarrow P_{\bigcap_{m = 1}^{\infty}U_{m}}(x_{1})$, and $z_{n} \rightarrow P_{\bigcap_{m = 1}^{\infty}U_{m}}(x_{1})$, as $n \rightarrow \infty$.

Following from the results of Step 2 and Step 5, $x_{n} \rightarrow P_{\bigcap_{m = 1}^{\infty}U_{m}}(x_{1})$, as $n \rightarrow\infty$. And then $x_{n+1} - x_{n} \rightarrow0$, as $n \rightarrow\infty$.

Since $x_{n+1} \in V_{n+1}\subset U_{n+1}$, $\alpha_{n} \rightarrow0$, and $e_{n} \rightarrow0$, then

$$\begin{aligned} 0 \leq&\phi(x_{n+1}, y_{n})\leq\alpha_{n} \phi(x_{n+1}, x_{1})+ (1-\alpha_{n}) \phi(x_{n+1}, x_{n}+e_{n}) \\ =& \alpha_{n} \Vert x_{n+1} \Vert ^{2}+ \alpha_{n} \Vert x_{1} \Vert ^{2} - 2 \alpha_{n} \langle x_{n+1}, Jx_{1}\rangle \\ &{}+(1-\alpha_{n}) \Vert x_{n+1} \Vert ^{2} + (1-\alpha_{n}) \Vert x_{n}+e_{n} \Vert ^{2} -2 (1-\alpha _{n}) \bigl\langle x_{n+1}, J(x_{n}+e_{n}) \bigr\rangle \\ =& \Vert x_{n+1} \Vert ^{2} - \alpha_{n} \Vert x_{1} \Vert ^{2} - (1-\alpha_{n}) \Vert x_{n}+e_{n} \Vert ^{2} \\ &{}+2\alpha_{n}\langle x_{1}-x_{n+1}, Jx_{1}\rangle + 2 (1-\alpha_{n}) \bigl\langle x_{n}+e_{n}-x_{n+1}, J(x_{n}+e_{n}) \bigr\rangle \\ \leq& \bigl( \Vert x_{n+1} \Vert ^{2}- \Vert x_{n}+e_{n} \Vert ^{2} \bigr)+ \alpha_{n} \bigl( \Vert x_{n}+e_{n} \Vert ^{2} - \Vert x_{1} \Vert ^{2} \bigr)+2 \alpha_{n} \Vert x_{1} \Vert \Vert x_{n+1}-x_{1} \Vert \\ &{}+2(1-\alpha_{n}) \Vert x_{n}+e_{n} \Vert \Vert x_{n}+e_{n}-x_{n+1} \Vert \rightarrow0, \end{aligned}$$

as $n \rightarrow\infty$. Lemma 2.4 implies that $x_{n+1} - y_{n}\rightarrow0$ and then $y_{n} \rightarrow P_{\bigcap_{m = 1}^{\infty }U_{m}}(x_{1})$ as $n \rightarrow\infty$.

Since $x_{n+1} \in V_{n+1}\subset U_{n+1}$ and $\beta_{n} \rightarrow0$, then

$$0 \leq\phi(x_{n+1}, z_{n})\leq\beta_{n} \phi(x_{n+1}, x_{1})+ (1-\beta_{n}) \phi(x_{n+1}, y_{n})\rightarrow0. $$

Lemma 2.4 implies that $x_{n+1} - z_{n}\rightarrow0$ and then $z_{n} \rightarrow P_{\bigcap_{m = 1}^{\infty}U_{m}}(x_{1})$ as $n \rightarrow \infty$.

*Step* 7. $P_{\bigcap_{m = 1}^{\infty}U_{m}}(x_{1}) \in(\bigcap_{i = 1}^{\infty}A_{i}^{-1}0) \cap(\bigcap_{i = 1}^{\infty}\operatorname{Fix}(B_{i}))$.

First, we shall show that $P_{\bigcap_{m = 1}^{\infty}U_{m}}(x_{1}) \in \bigcap_{i = 1}^{\infty}A_{i}^{-1}0$.

From (3.4) and Lemma 2.5, for $\forall q \in(\bigcap_{i = 1}^{\infty }A_{i}^{-1}0)\cap(\bigcap_{i = 1}^{\infty}\operatorname{Fix}(B_{i}))$, we have

$$\begin{aligned} \phi(q, y_{n}) \leq&\alpha_{n} \phi(q, x_{1})+ (1-\alpha _{n})\sum_{i = 1}^{\infty} a_{n,i}\phi \bigl(q, (J+r_{n,i}A_{i})^{-1}J(x_{n}+e_{n}) \bigr) \\ \leq&\alpha_{n} \phi(q, x_{1}) \\ &{}+ (1-\alpha_{n}) \sum_{i = 1}^{\infty} a_{n,i} \bigl[ \phi(q, x_{n}+e_{n})-\phi \bigl((J+r_{n,i}A_{i})^{-1}J(x_{n}+e_{n}),x_{n}+e_{n} \bigr) \bigr]. \end{aligned}$$

Thus

$$\begin{aligned}& (1-\alpha_{n})\sum_{i = 1}^{\infty} a_{n,i}\phi \bigl((J+r_{n,i}A_{i})^{-1}J(x_{n}+e_{n}),x_{n}+e_{n} \bigr) \\& \quad \leq\alpha_{n} \phi(q, x_{1})-\phi(q, y_{n})+ (1-\alpha_{n})\phi(q, x_{n}+e_{n}) \\& \quad = \alpha_{n} \bigl[\phi(q, x_{1})- \phi(q, x_{n}+e_{n}) \bigr]+ \bigl[\phi(q, x_{n}+e_{n})- \phi(q, y_{n}) \bigr] \\& \quad \leq\alpha_{n} \bigl[\phi(q, x_{1})- \phi(q, x_{n}+e_{n}) \bigr]+ \bigl( \Vert x_{n}+e_{n} \Vert ^{2}- \Vert y_{n} \Vert ^{2} \bigr)+2 \Vert q \Vert \bigl\Vert J(x_{n}+e_{n})-Jy_{n} \bigr\Vert . \end{aligned}$$

Since $\alpha_{n} \rightarrow0$, then $\sum_{i = 1}^{\infty} a_{n,i}\phi((J+r_{n,i}A_{i})^{-1}J(x_{n}+e_{n}),x_{n}+e_{n}) \rightarrow0$, which implies from Lemma 2.4 that $(J+r_{n,i}A_{i})^{-1}J(x_{n}+e_{n})- (x_{n}+e_{n}) \rightarrow0$, as $n \rightarrow\infty$. Thus $(J+r_{n,i}A_{i})^{-1}J(x_{n}+e_{n}) \rightarrow P_{\bigcap_{m = 1}^{\infty }U_{m}}(x_{1})$, as $n \rightarrow\infty$.

Let $u_{n,i} = (J+r_{n,i}A_{i})^{-1}J(x_{n}+e_{n})$, then $Ju_{n,i} + r_{n,i}A_{i}u_{n,i} = J(x_{n}+e_{n})$. Since $u_{n,i} \rightarrow P_{\bigcap _{m = 1}^{\infty}U_{m}}(x_{1})$, $x_{n} \rightarrow P_{\bigcap_{m = 1}^{\infty}U_{m}}(x_{1})$, $e_{n} \rightarrow0$, and $\inf_{n}r_{n,i} > 0$, then $A_{i}u_{n,i} \rightarrow0$ for $i \in N$, as $n \rightarrow\infty $. Using Lemma 2.2, $P_{\bigcap_{m = 1}^{\infty}U_{m}}(x_{1}) \in\bigcap_{i = 1}^{\infty}A_{i}^{-1}0$.

Next, we shall show that $P_{\bigcap_{m = 1}^{\infty}U_{m}}(x_{1})\in \bigcap_{i = 1}^{\infty}\operatorname{Fix}(B_{i})$.

Since $z_{n} = J^{-1}[ \beta_{n}Jx_{1} + (1-\beta_{n})\sum_{i = 1}^{\infty }b_{i}JB_{i}y_{n}]$, then $Jz_{n} - Jx_{n} = \beta_{n}(Jx_{1}-Jx_{n})+(1-\beta _{n})(\sum_{i = 1}^{\infty}b_{i}JB_{i}y_{n} - Jx_{n})$. Since both *J* and $J^{-1}$ are uniformly continuous on each bounded subset of *X*, $z_{n} \rightarrow P_{\bigcap_{m = 1}^{\infty}U_{m}}(x_{1})$, $x_{n} \rightarrow P_{\bigcap_{m = 1}^{\infty}U_{m}}(x_{1})$, and $\beta_{n} \rightarrow0$, then $\sum_{i = 1}^{\infty}b_{i}JB_{i}y_{n} - Jx_{n} \rightarrow0$, which implies that $J^{-1}(\sum_{i = 1}^{\infty}b_{i}JB_{i}y_{n}) \rightarrow P_{\bigcap_{m = 1}^{\infty}U_{m}}(x_{1})$, as $n \rightarrow\infty$.

The following proof is the same as the corresponding part in Step 7 of Theorem 3.1.

This completes the proof. □

### Theorem 3.3

*Suppose that* $\{x_{n}\}$ *is generated by the following iterative scheme*:

3.5 $$ \textstyle\begin{cases} x_{1} \in X,\qquad e_{1} \in X, \\ y_{n} = J^{-1}[\alpha_{n}Jx_{1}+(1-\alpha_{n})\sum_{i = 1}^{\infty} a_{n,i}J(J+r_{n,i}A_{i})^{-1}J(x_{n}+e_{n})], \\ z_{n} = J^{-1}[\beta_{n}Jx_{n}+(1-\beta_{n})\sum_{i = 1}^{\infty} b_{i}JB_{i}y_{n}], \\ U_{1} = X = V_{1}, \\ U_{n+1} = \{v \in U_{n}: \phi(v,y_{n}) \leq\alpha_{n} \phi(v,x_{1}) + (1-\alpha_{n})\phi(v,x_{n}+e_{n}), \\ \hphantom{U_{n+1} ={}}\phi(v,z_{n}) \leq\beta_{n} \phi(v,x_{n}) + (1-\beta_{n})\phi(v,y_{n})\}, \\ V_{n+1} = \{v \in U_{n+1}: \|x_{1} - v\|^{2} \leq\|P_{U_{n+1}}(x_{1})- x_{1}\| ^{2}+\lambda_{n+1}\}, \\ x_{n+1} \in V_{n+1},\quad n \in N. \end{cases} $$

*If* $0 \leq \sup_{n}\beta_{n} < 1$ *and* $\alpha_{n} \rightarrow0$, *then* $x_{n} \rightarrow P_{\bigcap_{m = 1}^{\infty}U_{m}}(x_{1}) \in(\bigcap_{i = 1}^{\infty}A_{i}^{-1}0)\cap(\bigcap_{i = 1}^{\infty}\operatorname{Fix}(B_{i}))$, *as* $n \rightarrow\infty$.

### Theorem 3.4

*Suppose that* $\{x_{n}\}$ *is generated by the following iterative scheme*:

3.6 $$ \textstyle\begin{cases} x_{1} \in X,\qquad e_{1} \in X, \\ y_{n} = J^{-1}[\alpha_{n}Jx_{n}+(1-\alpha_{n})\sum_{i = 1}^{\infty} a_{n,i}J(J+r_{n,i}A_{i})^{-1}J(x_{n}+e_{n})], \\ z_{n} = J^{-1}[\beta_{n}Jx_{1}+(1-\beta_{n})\sum_{i = 1}^{\infty} b_{i}JB_{i}y_{n}], \\ U_{1} = X = V_{1}, \\ U_{n+1} = \{v \in U_{n}: \phi(v,y_{n}) \leq\alpha_{n} \phi(v,x_{n}) + (1-\alpha_{n})\phi(v,x_{n}+e_{n}), \\ \hphantom{U_{n+1} ={}}\phi(v,z_{n}) \leq\beta_{n} \phi(v,x_{1}) + (1-\beta_{n})\phi(v,y_{n})\}, \\ V_{n+1} = \{v \in U_{n+1}: \|x_{1} - v\|^{2} \leq\|P_{U_{n+1}}(x_{1})- x_{1}\| ^{2}+\lambda_{n+1}\}, \\ x_{n+1} \in V_{n+1},\quad n \in N. \end{cases} $$

*If* $0 \leq \sup_{n}\alpha_{n} < 1$ *and* $\beta_{n} \rightarrow0$, *then* $x_{n} \rightarrow P_{\bigcap_{m = 1}^{\infty}U_{m}}(x_{1}) \in(\bigcap_{i = 1}^{\infty}A_{i}^{-1}0)\cap(\bigcap_{i = 1}^{\infty}\operatorname{Fix}(B_{i}))$, *as* $n \rightarrow\infty$.

### Remark 3.5

The main difference between ours and [8] is that: in [8], in each step *n*, countable sets $V_{n+1,i}$ and $W_{n+1,i}$ are needed to be evaluated, but in our paper, in each step *n*, two sets $U_{n+1}$ and $V_{n+1}$ are enough. This difference leads to some different techniques for proving the main results.

### Corollary 3.6

*If* *X* *reduces to a Hilbert space* *H*, *then* (3.1) *becomes as follows*:

3.7 $$ \textstyle\begin{cases} x_{1} \in H,\qquad e_{1} \in H, \\ y_{n} = \alpha_{n}x_{n}+(1-\alpha_{n})\sum_{i = 1}^{\infty} a_{n,i}(I+r_{n,i}A_{i})^{-1}(x_{n}+e_{n}), \\ z_{n} = \beta_{n}x_{n}+(1-\beta_{n})\sum_{i = 1}^{\infty} b_{i}B_{i}y_{n}, \\ U_{1} = H = V_{1}, \\ U_{n+1} = \{v \in U_{n}: \|v - y_{n}\|^{2} \leq\alpha_{n} \|v - x_{n}\|^{2} + (1-\alpha_{n})\|v-x_{n}-e_{n}\|^{2}, \\ \hphantom{U_{n+1} ={}}\|v - z_{n}\|^{2} \leq\beta_{n} \|v - x_{n}\|^{2} + (1-\beta_{n})\|v-y_{n}\|^{2}\} , \\ V_{n+1} = \{v \in U_{n+1}: \|x_{1} - v\|^{2} \leq\|P_{U_{n+1}}(x_{1})- x_{1}\| ^{2}+\lambda_{n+1}\}, \\ x_{n+1} \in V_{n+1},\quad n \in N. \end{cases} $$

*Similarly*, *we can get the special forms of* (3.4), (3.5), *and* (3.6) *in the frame of Hilbert space H*.

### Corollary 3.7

*If*, *further*, $r_{n,i} \equiv r_{n}$, $A_{i} \equiv A$, *and* $B_{i} \equiv B$, *then we can get a special case for* (3.7):

3.8 $$ \textstyle\begin{cases} x_{1} \in H,\qquad e_{1} \in H, \\ y_{n} = \alpha_{n}x_{n}+(1-\alpha_{n})(I+r_{n}A)^{-1}(x_{n}+e_{n}), \\ z_{n} = \beta_{n}x_{n}+(1-\beta_{n})By_{n}, \\ U_{1} = H = V_{1}, \\ U_{n+1} = \{v \in U_{n}: \|v - y_{n}\|^{2} \leq\alpha_{n} \|v - x_{n}\|^{2} + (1-\alpha_{n})\|v-x_{n}-e_{n}\|^{2}, \\ \hphantom{U_{n+1} ={}}\|v - z_{n}\|^{2} \leq\beta_{n} \|v - x_{n}\|^{2} + (1-\beta_{n})\|v-y_{n}\|^{2}\} , \\ V_{n+1} = \{v \in U_{n+1}: \|x_{1} - v\|^{2} \leq\|P_{U_{n+1}}(x_{1})- x_{1}\| ^{2}+\lambda_{n+1}\}, \\ x_{n+1} \in V_{n+1}, \quad n \in N, \end{cases} $$

*where* *A* *is maximal monotone*, *B* *is weakly relatively non*-*expansive*, *and* $\{r_{n}\}\subset[0, +\infty)$ *satisfies* $\inf_{n}r_{n} > 0$.

### Corollary 3.8

*If*, *in Corollary *3.7, $\alpha_{n} \equiv0$, *then* (3.8) *can be further simplified as follows*:

3.9 $$ \textstyle\begin{cases} x_{1} \in H, \qquad e_{1} \in H, \\ y_{n} = (I+r_{n}A)^{-1}(x_{n}+e_{n}), \\ z_{n} = \beta_{n}x_{n}+(1-\beta_{n})By_{n}, \\ U_{1} = H = V_{1}, \\ U_{n+1} = \{v \in U_{n}: \|v - y_{n}\|\leq\|v - x_{n} - e_{n}\|, \\ \hphantom{U_{n+1} ={}}\|v - z_{n}\|^{2} \leq\beta_{n} \|v - x_{n}\|^{2} + (1-\beta_{n})\|v-y_{n}\|^{2}\} , \\ V_{n+1} = \{v \in U_{n+1}: \|x_{1} - v\|^{2} \leq\|P_{U_{n+1}}(x_{1})- x_{1}\| ^{2}+\lambda_{n+1}\}, \\ x_{n+1} \in V_{n+1},\quad n \in N. \end{cases} $$

### Remark 3.9

Comparing (3.9) and (1.4), we may find that they are different due to different construction of $U_{n+1}$. This indicates again that (3.1) is different from (1.3).

### Remark 3.10

Choose $H = (-\infty,+\infty)$, $Ax = 2x$, and $Bx = x$ for $x \in(-\infty,+\infty)$. Let $e_{n} = \beta_{n} = \lambda_{n} = \frac {1}{n}$ and $r_{n} = 2^{n-1}$ for $n \in N$. Then *A* is maximal monotone and *B* is weakly relatively non-expansive. Moreover, $A^{-1}0 \cap \operatorname{Fix}(B) = \{0\}$.

### Corollary 3.11

*Take the example in Remark *3.10. *We can choose the following three iterative sequences* $\{x_{n}\}$ *among infinite choices by iterative scheme* (3.9).

3.10 $$\begin{aligned}& \textstyle\begin{cases} x_{1} = 1,\qquad x_{2} = 1-\frac{\sqrt{2}}{2}, \\ y_{n} = \frac{x_{n}+e_{n}}{1+2r_{n}},\quad n \in N, \\ w_{n} = \min_{m \leq n} (1+r_{m})y_{m}, \quad n \in N, \\ x_{n+1}= x_{1}-\sqrt{(x_{1}-w_{n})^{2}+\lambda_{n+1}},\quad n \in N \setminus\{1\}, \end{cases}\displaystyle \end{aligned}$$

3.11 $$\begin{aligned}& \textstyle\begin{cases} x_{1} = 1, \\ y_{n} = \frac{x_{n}+e_{n}}{1+2r_{n}},\quad n \in N, \\ x_{n+1}=(1+r_{n})y_{n},\quad n \in N, \end{cases}\displaystyle \end{aligned}$$

*and*

3.12 $$ \textstyle\begin{cases} x_{1} = 1,\qquad x_{2} = \frac{7}{6}-\frac{\sqrt{2}}{4}, \\ y_{n} = \frac{x_{n}+e_{n}}{1+2r_{n}},\quad n \in N, \\ w_{n} = \min_{m \leq n} (1+r_{m})y_{m},\quad n \in N, \\ x_{n+1}= \frac{x_{1}+w_{n}-\sqrt{(x_{1}-w_{n})^{2}+\lambda_{n+1}}}{2},\quad n \in N \setminus\{1\}. \end{cases} $$

*Then* $\{x_{n}\}$ *generated by* (3.10), (3.11), *and* (3.12) *converges strongly to* $0 \in A^{-1}0\cap \operatorname{Fix}(B)$, *as* $n\rightarrow\infty$.

### Proof

We can easily see from iterative scheme (3.9) that

3.13 $$ y_{n} = \frac{x_{n}+e_{n}}{1+2r_{n}} \quad \mbox{for }n \in N, $$

and

3.14 $$ z_{n} = \beta_{n}x_{n}+(1-\beta_{n})y_{n}\quad \mbox{for }n \in N. $$

From (3.14), we can see that $(v - z_{n})^{2} \leq\beta_{n} (v - x_{n})^{2} + (1-\beta_{n})(v-y_{n})^{2}$ is always true for $v \in(-\infty, +\infty)$. Then we can simplify $U_{n+1}$ and $V_{n+1}$ as follows:

3.15 $$ U_{n+1} = U_{n} \cap \bigl\{ v \in(-\infty, +\infty): 2(x_{n}+e_{n}-y_{n})v \leq (x_{n}+e_{n})^{2}-y_{n}^{2} \bigr\} \quad \mbox{for }n \in N, $$

and

3.16 $$\begin{aligned} \begin{aligned}[b] &V_{n+1} = U_{n+1} \cap \bigl[x_{1} - \sqrt{ \bigl(x_{1}-P_{U_{n+1}}(x_{1}) \bigr)^{2}+ \lambda _{n+1}}, x_{1} + \sqrt{ \bigl(x_{1}-P_{U_{n+1}}(x_{1}) \bigr)^{2}+\lambda_{n+1}} \bigr] \\ &\quad \mbox{for }n \in N. \end{aligned} \end{aligned}$$

Next, we split the proof into three parts.

*Part* 1. We shall show that both $\{x_{n}\}$ and $\{y_{n}\}$ generated by (3.10) converge strongly to $0 \in A^{-1}0\cap \operatorname{Fix}(B)$, as $n\rightarrow\infty$.

By using inductive method, we first show that the following is true:

3.17 $$ \textstyle\begin{cases} x_{1} = 1,\qquad x_{2} = 1 - \frac{\sqrt{2}}{2}, \\ 0 < (1+r_{n+1})y_{n+1} < 1,\quad n \in N, \\ U_{1} = (-\infty, +\infty) = V_{1}, \\ U_{2} = (-\infty,\frac{4}{3}],\qquad V_{2} = [1-\frac{\sqrt{2}}{2}, \frac {4}{3}], \\ U_{n+1} = (-\infty, w_{n}],\quad n \in N\setminus\{1\}, \\ V_{n+1} = [x_{1} - \sqrt{(x_{1}-w_{n})^{2}+\lambda_{n+1}},w_{n}],\quad n \in N\setminus\{1\}, \\ \mbox{we may choose }x_{n+1} = x_{1} - \sqrt{(x_{1}-w_{n})^{2}+\lambda_{n+1}}, \quad n \in N \setminus\{1\}. \end{cases} $$

In fact, if $n = 1$, $y_{1} = \frac{x_{1}+e_{1}}{1+2r_{1}} = \frac{2}{3}$. Since $(x_{1}+e_{1})-y_{1} = 2r_{1}y_{1} = 2y_{1} = \frac{4}{3} > 0$, then from (3.15), $U_{2} = (-\infty, +\infty)\cap(-\infty,(1+r_{1})y_{1}]= (-\infty ,\frac{4}{3}]$. Thus $P_{U_{2}}(x_{1}) = x_{1}$. From (3.16), $V_{2} = U_{2} \cap[1-\frac {\sqrt{2}}{2}, 1+\frac{\sqrt{2}}{2}] = [1-\frac{\sqrt{2}}{2}, \frac{4}{3}]$. So, we may choose $x_{2} = 1 - \frac{\sqrt{2}}{2}$.

If $n = 2$, $y_{2} = \frac{x_{2}+e_{2}}{1+2r_{2}} = \frac{3}{10}-\frac{\sqrt {2}}{10}$ and $w_{2} = \min\{(1+r_{1})y_{1}, (1+r_{2})y_{2}\} = \frac{9-3\sqrt {2}}{10} = (1+r_{2})y_{2}$. It is easy to see that $0 < (1+r_{2})y_{2} < 1$, and then $x_{2} + e_{2} - y_{2} = 2r_{2}y_{2} > 0$. From (3.15), $U_{3} = U_{2} \cap(-\infty, 3y_{2}] = [-\infty, \frac{4}{3}] \cap(-\infty, \frac {9-3\sqrt{2}}{10}] = (-\infty, w_{2}]$, and then $P_{U_{3}}(x_{1}) = w_{2} = \frac{9-3\sqrt{2}}{10}$. From (3.16), $V_{3} = U_{3} \cap[x_{1}-\sqrt {(x_{1}-w_{2})^{2} + \lambda_{3}}, x_{1}+\sqrt{(x_{1}-w_{2})^{2} + \lambda_{3}}] = [1-\sqrt{(\frac{1+3\sqrt{2}}{10})^{2}+\frac{1}{3}},\frac{9-3\sqrt {2}}{10}] = [x_{1}-\sqrt{(x_{1}-w_{2})^{2} + \lambda_{3}},w_{2}]$. Then we may choose $x_{3} = x_{1}-\sqrt{(x_{1}-w_{2})^{2} + \lambda_{3}}$.

Suppose that (3.17) is true for $n = k$. We now begin the discussion for $n = k+1$.

Since $0 < (1+r_{k+1})y_{k+1} < 1$, then $x_{k+1}+e_{k+1}-y_{k+1} = 2r_{k+1}y_{k+1} > 0$. From (3.15) and (3.13), $U_{k+2} = U_{k+1} \cap(-\infty, (1+r_{k+1})y_{k+1}] = (-\infty, w_{k+1}]$, and then $P_{U_{k+2}}(x_{1}) = w_{k+1}$.

Note that $w_{k+1} < 1 = x_{1} < x_{1} + \sqrt{(x_{1}-w_{k+1})^{2}+\lambda _{k+2}}$ and $\sqrt{(x_{1}-w_{k+1})^{2}+\lambda_{k+2}}> x_{1}-w_{k+1}> 0$, then from (3.16) we know that

$$V_{k+2} = \bigl[x_{1} - \sqrt{(x_{1}-w_{k+1})^{2}+ \lambda_{k+2}},w_{k+1} \bigr]. $$

Then we may choose

$$x_{k+2} = x_{1} - \sqrt{(x_{1}-w_{k+1})^{2}+ \lambda_{k+2}}. $$

Since $y_{k+2} = \frac{x_{k+2}+e_{k+2}}{1+2r_{k+2}} = \frac {x_{k+2}}{1+2^{k+2}}+\frac{1}{(k+2)(1+2^{k+2})}$, then $(1+r_{k+2})y_{k+2} = \frac{1+r_{k+2}}{1+2r_{k+2}}(x_{k+2}+e_{k+2})$. Note that

$$\begin{aligned} (1+r_{k+2})y_{k+2} > 0&\quad \Longleftrightarrow\quad x_{k+2}+e_{k+2} > 0 \\ &\quad\Longleftrightarrow\quad 1 + \frac{1}{k+2} > \sqrt{(1-w_{k+1})^{2}+ \lambda _{k+2}} \\ &\quad\Longleftrightarrow\quad 1+\frac{2}{k+2}+\frac{1}{(k+2)^{2}} > (1-w_{k+1})^{2}+\frac{1}{k+2} \\ &\quad\Longleftrightarrow\quad 1+\frac{1}{k+2}+\frac{1}{(k+2)^{2}} > (1-w_{k+1})^{2}. \end{aligned}$$

This is obviously true. Then $(1+r_{k+2})y_{k+2} > 0$. Since

$$\begin{aligned} x_{k+2}+\frac{1}{k+2} =& 1-\sqrt{(1-w_{k+1})^{2}+ \frac {1}{k+2}}+\frac{1}{k+2} < w_{k+1}+ \frac{1}{k+2} \\ < & 1+ \frac{1}{k+2} < \frac{1+2^{k+2}}{1+2^{k+1}}= \frac{1+2r_{k+2}}{1+r_{k+2}}, \end{aligned}$$

then $(1+r_{k+2})y_{k+2}= \frac{1+r_{k+2}}{1+2r_{k+2}} (x_{k+2}+e_{k+2}) < 1$.

By now, we have proved that (3.17) is true.

In this part, it is left to prove that $x_{n} \rightarrow0$, $y_{n} \rightarrow0$, as $n \rightarrow\infty$.

From (3.17), $\{(1+r_{n})y_{n}\}$ is bounded, which implies that $\{w_{n}\}$ is bounded. Thus $\{x_{n}\}$ is bounded. Let $\{x_{n_{i}}\}$ be any subsequence of $\{x_{n}\}$ such that $\lim_{i \rightarrow\infty}x_{n_{i}} = a$. Then $w_{n_{i}} \rightarrow a$ and $y_{n_{i}}\rightarrow0$ as $i \rightarrow\infty$. Since $0 < w_{n_{i}} \leq(1+r_{n_{i}})y_{n_{i}} < 1$, then $0 \leq a \leq \lim_{i \rightarrow\infty}(1+r_{n_{i}})y_{n_{i}}\leq 1$. That is, $0 \leq a \leq \lim_{i \rightarrow\infty }r_{n_{i}}y_{n_{i}}\leq1$. From the fact that $2r_{n}y_{n} = x_{n} + e_{n} - y_{n}$, we have $\lim_{i \rightarrow\infty}(1+r_{n_{i}})y_{n_{i}} = \frac{a}{2}$. By now, we know that $0 \leq a \leq\frac{a}{2} \leq1$, then $a = 0$. This means that each strongly convergent subsequence of $\{x_{n}\}$ converges strongly to 0. Thus $x_{n} \rightarrow0 \in A^{-1}0 \cap \operatorname{Fix}(B)$, as $n \rightarrow\infty$. And then $y_{n} \rightarrow0$, $w_{n} \rightarrow0$, as $n \rightarrow\infty$.

*Part* 2. We shall show that both $\{x_{n}\}$ and $\{y_{n}\}$ generated by (3.11) converge strongly to $0 \in A^{-1}0\cap \operatorname{Fix}(B)$, as $n\rightarrow\infty$.

First, we shall use inductive method to show that the following is true:

3.18 $$ \textstyle\begin{cases} x_{1} = 1, \\ 0 < (1+r_{n+1})y_{n+1} < (1+ r_{n})y_{n}, \quad n \in N, \\ \frac{1+2^{n+1}}{(n+2)2^{n+1}}< (1+r_{n+1})y_{n+1}< 1,\quad n \in N\setminus \{1\}, \\ U_{1} = (-\infty, +\infty) = V_{1},\qquad V_{2} = [1-\frac{\sqrt{2}}{2}, \frac {4}{3}], \qquad V_{3} = [1-\frac{\sqrt{3}}{3}, \frac{11}{10}], \\ U_{n+1} = (-\infty,(1+r_{n})y_{n}],\quad n \in N\setminus\{1\}, \\ V_{n+1} = [x_{1} - \sqrt{[x_{1}-(1+r_{n})y_{n}]^{2}+\lambda_{n+1}}, (1+r_{n})y_{n}],\quad n \in N\setminus\{1, 2\}, \\ \mbox{we may choose }x_{n+1} = (1+r_{n})y_{n},\quad n \in N. \end{cases} $$

In fact, if $n = 1$, $y_{1} = \frac{x_{1}+e_{1}}{1+2r_{1}} = \frac{2}{3}$. Since $(x_{1}+e_{1})-y_{1} = 2r_{1}y_{1} = 2y_{1} = \frac{4}{3} > 0$, then from (3.15), $U_{2} = (-\infty, +\infty)\cap(-\infty,(1+r_{1})y_{1}]= (-\infty ,\frac{4}{3}]$. Thus $P_{U_{2}}(x_{1}) = x_{1}$. From (3.16), $V_{2} = U_{2} \cap[1-\frac {\sqrt{2}}{2}, 1+\frac{\sqrt{2}}{2}] = [1-\frac{\sqrt{2}}{2}, \frac{4}{3}]$. Then we may choose $x_{2} = (1+r_{1})y_{1} = \frac{4}{3}$.

If $n = 2$, $y_{2} = \frac{x_{2}+e_{2}}{1+2r_{2}} = \frac{11}{30}$. It is easy to see that $0 < (1+r_{2})y_{2} = \frac{11}{10} < (1+r_{1})y_{1} = \frac {4}{3}$. From (3.15), $U_{3} = U_{2} \cap(-\infty, 3y_{2}] = (-\infty, \frac{11}{10}] = (-\infty, (1+r_{2})y_{2}]$, and then $P_{U_{3}}(x_{1}) = x_{1}$. From (3.16), $V_{3} = U_{3} \cap[1-\frac{\sqrt{3}}{3}, 1+\frac {\sqrt{3}}{3}]= [1-\frac{\sqrt{3}}{3}, \frac{11}{10}]$. Then we may choose $x_{3} = (1+r_{2})y_{2} = \frac{11}{10}$. Thus $y_{3} = \frac {x_{3}+e_{3}}{1+2r_{3}} = \frac{43}{270}$. It is easy to check that $0 < (1+r_{3})y_{3} = \frac{43}{54}< \frac{11}{10} = (1+r_{2})y_{2}$ and $\frac {1+2^{3}}{(2+2)2^{3}} < (1+r_{3})y_{3} < 1$.

Suppose that (3.18) is true for $n = k$. Next, we show the result is true for $n = k+1$.

Since $0 < (1+r_{k+1})y_{k+1} < (1+ r_{k})y_{k} < 1$, then (3.15) implies that $U_{k+2} = U_{k+1} \cap(-\infty, (1+r_{k+1})y_{k+1}] = (-\infty,(1+r_{k+1})y_{k+1}]$ and $P_{U_{k+2}}(x_{1}) = (1+r_{k+1})y_{k+1}$.

Note that $(1+r_{k+1})y_{k+1} < 1 = x_{1} < x_{1} + \sqrt {[x_{1}-(1+r_{k+1})y_{k+1}]^{2}+\lambda_{k+2}}$ and $x_{1} - \sqrt{[x_{1}-(1+r_{k+1})y_{k+1}]^{2}+\lambda _{k+2}}<(1+r_{k+1})y_{k+1}$. Then, from (3.16), we know that

$$V_{k+2} = \bigl[x_{1} - \sqrt{ \bigl[x_{1}-{(1+r_{k+1})y_{k+1} \bigr]^{2}+\lambda_{k+2}}}, (1+r_{k+1})y_{k+1} \bigr]. $$

Thus we may choose

$$x_{k+2} = (1+r_{k+1})y_{k+1}. $$

And then, $y_{k+2} = \frac{x_{k+2}+e_{k+2}}{1+2r_{k+2}} = \frac {x_{k+2}}{1+2^{k+2}}+\frac{1}{(k+2)(1+2^{k+2})}$. So $(1+r_{k+2})y_{k+2} = \frac{1+r_{k+2}}{1+2r_{k+2}}(x_{k+2}+e_{k+2})$. Note that

$$(1+r_{k+2})y_{k+2} > 0\quad \Longleftrightarrow\quad x_{k+2}+e_{k+2} > 0 \quad \Longleftrightarrow\quad (1+r_{k+1})y_{k+1} + \frac {1}{k+2} > 0, $$

which is obviously true from the assumption. Thus $(1+r_{k+2})y_{k+2} > 0$.

Since $(1+r_{k+1})y_{k+1}< 1$, then $\frac {1+2^{k+1}}{1+2^{k+2}}[(1+r_{k+1})y_{k+1}+\frac{1}{k+2}]<\frac {1+2^{k+1}}{1+2^{k+2}} \frac{k+3}{k+2}< 1$. Thus

$$(1+r_{k+2})y_{k+2} = (1+r_{k+2}) \frac {x_{k+2}+e_{k+2}}{1+2r_{k+2}} = \frac{1+2^{k+1}}{1+2^{k+2}} \biggl[(1+r_{k+1})y_{k+1}+\frac{1}{k+2} \biggr]< 1. $$

Note that

$$\begin{aligned} &(1+r_{k+2})y_{k+2}< (1+r_{k+1})y_{k+1} \\ &\quad \Longleftrightarrow\quad \frac {1+r_{k+2}}{1+2r_{k+2}}(x_{k+2}+e_{k+2}) < (1+r_{k+1})y_{k+1} \\ &\quad\Longleftrightarrow \quad \frac {1+2^{k+1}}{1+2^{k+2}} \biggl[(1+r_{k+1})y_{k+1}+ \frac{1}{k+2} \biggr] < (1+r_{k+1})y_{k+1} \\ &\quad\Longleftrightarrow\quad \frac {2^{k+2}-2^{k+1}}{1+2^{k+2}}(1+r_{k+1})y_{k+1}> \frac {1+2^{k+1}}{(k+2)(1+2^{k+2})} \\ &\quad\Longleftrightarrow\quad (1+r_{k+1})y_{k+1}> \frac{1+2^{k+1}}{(k+2)2^{k+1}}, \end{aligned}$$

which is true from the assumption.

Compute the following:

$$\begin{aligned} (1+r_{k+2})y_{k+2} =& \frac {1+r_{k+2}}{1+2r_{k+2}}(x_{k+2}+e_{k+2}) \\ =& \frac {1+2^{k+1}}{1+2^{k+2}} \biggl[(1+r_{k+1})y_{k+1}+ \frac{1}{k+2} \biggr] \\ >& \frac{1+2^{k+1}}{1+2^{k+2}} \biggl[\frac{1+2^{k+1}}{(k+2)2^{k+1}}+\frac {1}{k+2} \biggr] \\ =& \frac{1+2^{k+1}}{(k+2)2^{k+1}} > \frac{1+2^{k+2}}{(k+3)2^{k+2}}. \end{aligned}$$

By now, we have proved that (3.18) is true.

In this part, it is left to prove that $x_{n} \rightarrow0$, $y_{n} \rightarrow0$, as $n \rightarrow\infty$.

Since $\{(1+r_{n})y_{n}\}$ is decreasing and bounded in $(0,1)$, then $\lim_{n \rightarrow\infty}(1+r_{n})y_{n} = \lim_{n \rightarrow\infty}x_{n} = a$. Coming back to (3.13), we know that $r_{n}y_{n} \rightarrow0$, as $n \rightarrow\infty$. Then $y_{n} \rightarrow0$, and then $x_{n} \rightarrow0$, as $n \rightarrow\infty$.

*Part* 3. We shall show that both $\{x_{n}\}$ and $\{y_{n}\}$ generated by (3.12) converge strongly to $0 \in A^{-1}0\cap \operatorname{Fix}(B)$, as $n\rightarrow\infty$.

First, we shall use inductive method to show that the following is true:

3.19 $$ \textstyle\begin{cases} x_{1} = 1, \qquad x_{2} = \frac{7}{6} - \frac{\sqrt{2}}{4}, \\ 0 < (1+r_{n+1})y_{n+1} < 1, \quad n \in N, \\ U_{1} = (-\infty, +\infty) = V_{1}, \\ U_{2} = (-\infty,\frac{4}{3}],\qquad V_{2} = [1-\frac{\sqrt{2}}{2}, \frac {4}{3}], \\ U_{n+1} = (-\infty, w_{n}],\quad n \in N\setminus\{1\}, \\ V_{n+1} = [x_{1} - \sqrt{(x_{1}-w_{n})^{2}+\lambda_{n+1}},w_{n}],\quad n \in N\setminus\{1\}, \\ \mbox{we may choose }x_{n+1} = \frac{x_{1} - \sqrt{(x_{1}-w_{n})^{2}+\lambda _{n+1}}+w_{n}}{2},\quad n \in N \setminus\{1\}. \end{cases} $$

In fact, if $n = 1$, $y_{1} = \frac{x_{1}+e_{1}}{1+2r_{1}} = \frac{2}{3}$. Since $(x_{1}+e_{1})-y_{1} = 2r_{1}y_{1} = 2y_{1} = \frac{4}{3} > 0$, then from (3.15), $U_{2} = (-\infty, +\infty)\cap(-\infty,(1+r_{1})y_{1}]= (-\infty ,\frac{4}{3}]$. Then $P_{U_{2}}(x_{1}) = x_{1}$. From (3.16), $V_{2} = U_{2} \cap[1-\frac {\sqrt{2}}{2}, 1+\frac{\sqrt{2}}{2}] = [1-\frac{\sqrt{2}}{2}, \frac{4}{3}]$. Thus we may choose $x_{2} = \frac{1-\frac{\sqrt{2}}{2}+\frac{4}{3}}{2} = \frac{7}{6} - \frac{\sqrt{2}}{4}$.

If $n = 2$, $y_{2} = \frac{x_{2}+e_{2}}{1+2r_{2}} = \frac{1}{3}-\frac{\sqrt {2}}{20}$ and $w_{2} = \min\{(1+r_{1})y_{1}, (1+r_{2})y_{2}\} = 1-\frac {3\sqrt{2}}{20}$. It is easy to see that $0 < (1+r_{2})y_{2} = 1 -\frac {3\sqrt{2}}{20}< 1$. Thus from (3.15), $U_{3} = U_{2} \cap(-\infty, 3y_{2}] = (-\infty, \frac{4}{3}] \cap(-\infty, 1-\frac{3\sqrt {2}}{20}] = (-\infty, w_{2}]$, and then $P_{U_{3}}(x_{1}) = 1-\frac{3\sqrt {2}}{20} = w_{2}$.

From (3.16), $V_{3} = U_{3} \cap[x_{1}-\sqrt{(x_{1}-w_{2})^{2} + \lambda_{3}}, x_{1}+\sqrt{(x_{1}-w_{2})^{2} + \lambda_{3}}] = [1-\sqrt{\frac{18}{400}+\frac {1}{3}}, 1- \frac{3\sqrt{2}}{20}] = [x_{1}-\sqrt{(x_{1}-w_{2})^{2} + \lambda _{3}},w_{2}]$. Then we may choose $x_{3} = \frac{x_{1}-\sqrt{(x_{1}-w_{2})^{2} + \lambda_{3}}+w_{2}}{2}= 1- \frac{3\sqrt{2}}{40} - \frac{\sqrt{1362}}{120}$. We can easily check that $0 < (1+r_{3})y_{3} = 5y_{3} = \frac{20}{27}-\frac {9\sqrt{2}+\sqrt{1362}}{216} < 1$.

Suppose that (3.19) is true for $n = k$. Next, we shall show that (3.19) is true for $n = k+1$.

Since $0 <(1+r_{k+1})y_{k+1}< 1$, then $x_{k+1}+e_{k+1}-y_{k+1} = 2r_{k+1}y_{k+1} > 0$. From (3.15), $U_{k+2} = U_{k+1} \cap(-\infty, (1+r_{k+1})y_{k+1}] = (-\infty, w_{k+1}]$, and $P_{U_{k+2}}(x_{1}) = w_{k+1}$. From (3.16), $V_{k+2} = U_{k+2} \cap[x_{1} - \sqrt {(x_{1}-w_{k+1})^{2}+\lambda_{k+2}},x_{1} + \sqrt{(x_{1}-w_{k+1})^{2}+\lambda_{k+2}}]$.

Note that $w_{k+1} < 1 = x_{1} < x_{1} + \sqrt{(x_{1}-w_{k+1})^{2}+\lambda _{k+2}}$ and $\sqrt{(x_{1}-w_{k+1})^{2}+\lambda_{k+2}}> x_{1}- w_{k+1}> 0$. Then $V_{k+2} = [x_{1} - \sqrt{(x_{1}-w_{k+1})^{2}+\lambda_{k+2}}, w_{k+1}]$. Thus we may choose

$$x_{k+2} = \frac{x_{1} - \sqrt{(x_{1}-w_{k+1})^{2}+\lambda_{k+2}}+w_{k+1}}{2}. $$

Note that

$$\begin{aligned} (1+r_{k+2})y_{k+2} > 0&\quad \Longleftrightarrow\quad x_{k+2}+e_{k+2} > 0 \\ &\quad\Longleftrightarrow\quad \frac{1-\sqrt{(1-w_{k+1})^{2}+\frac {1}{k+2}}+w_{k+1}}{2} + \frac{1}{k+2} > 0 \\ &\quad\Longleftrightarrow\quad \frac{1+w_{k+1}}{2} + \frac{1}{k+2} > \frac{\sqrt {(1-w_{k+1})^{2}+\frac{1}{k+2}}}{2} \\ &\quad\Longleftrightarrow\quad \biggl(\frac{k+4}{k+2} \biggr)^{2}+ \frac{2(k+4)}{k+2}w_{k+1} > 1-2w_{k+1}+\frac{1}{k+2} \\ &\quad\Longleftrightarrow\quad \biggl(\frac{k+4}{k+2} \biggr)^{2}+ \frac{12+4k}{k+2}w_{k+1} > \frac {k+3}{k+2}, \end{aligned}$$

which is obviously true since $(\frac{k+4}{k+2})^{2} > 1+\frac{1}{k+2}$. Then $(1+r_{k+2})y_{k+2} > 0$.

Moreover,

$$\begin{aligned} &(1+r_{k+2})y_{k+2}< 1 \\ &\quad\Longleftrightarrow\quad \frac {1+r_{k+2}}{1+2r_{k+2}} \biggl(x_{k+2}+\frac{1}{k+2} \biggr) < 1 \\ &\quad\Longleftrightarrow\quad \frac{1+w_{k+1}-\sqrt{(1-w_{k+1})^{2}+\frac {1}{k+2}}}{2}< \frac{1+2r_{k+2}}{1+r_{k+2}}- \frac{1}{k+2} \\ &\quad\Longleftrightarrow\quad 1+w_{k+1}< \sqrt{(1-w_{k+1})^{2}+ \frac{1}{k+2}}+\frac {2(1+2^{k+2})}{1+2^{k+1}}-\frac{2}{k+2} \\ &\quad\Longleftrightarrow\quad w_{k+1}< \sqrt{(1-w_{k+1})^{2}+ \frac{1}{k+2}}+\frac {1+3\cdot2^{k+1}}{1+2^{k+1}}-\frac{2}{k+2} \end{aligned}$$

which is true since $\frac{1+3\cdot2^{k+1}}{1+2^{k+1}}-\frac{2}{k+2} > 1$. Then $(1+r_{k+2})y_{k+2}= \frac{1+r_{k+2}}{1+2r_{k+2}} (x_{k+2}+e_{k+2}) < 1$.

By now, we have proved that (3.19) is true.

In this part, it is left to prove that $x_{n} \rightarrow0$, $y_{n} \rightarrow0$, as $n \rightarrow\infty$.

From (3.19), $\{(1+r_{n})y_{n}\}$ is bounded, which implies that $\{w_{n}\}$ is bounded. Then we can easily check that $\{x_{n}\}$ is bounded. Let $\{ x_{n_{i}}\}$ be any subsequence of $\{x_{n}\}$ such that $\lim_{i \rightarrow\infty}x_{n_{i}} = a$. Then $w_{n_{i}} \rightarrow a$ and $y_{n_{i}}\rightarrow0$ as $i \rightarrow\infty$. Since $2r_{n}y_{n} = x_{n} + e_{n} - y_{n}$, then $\lim_{i \rightarrow\infty}(1+r_{n_{i}})y_{n_{i}} = \frac {a}{2}$. Since $0 < w_{n_{i}} \leq(1+r_{n_{i}})y_{n_{i}} < 1$, then $0 \leq a \leq\frac{a}{2}\leq1$. Thus $a = 0$. This means that each strongly convergent subsequence of $\{x_{n}\}$ converges strongly to 0. Thus $x_{n} \rightarrow0$, as $n \rightarrow\infty$. And then $y_{n} \rightarrow 0$, $w_{n} \rightarrow0$, as $n \rightarrow\infty$.

This completes the proof. □

### Remark 3.12

**Figure 1:**
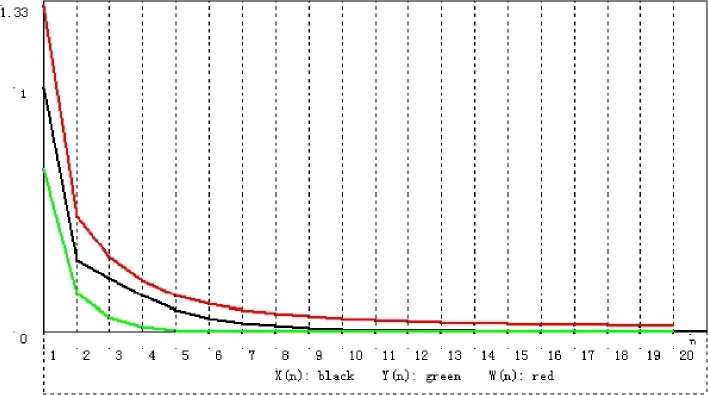
Convergence of $\{x_{n}\}$, $\{y_{n}\}$, and $\{w_{n}\}$ corresponding to Table 1

**Figure 2:**
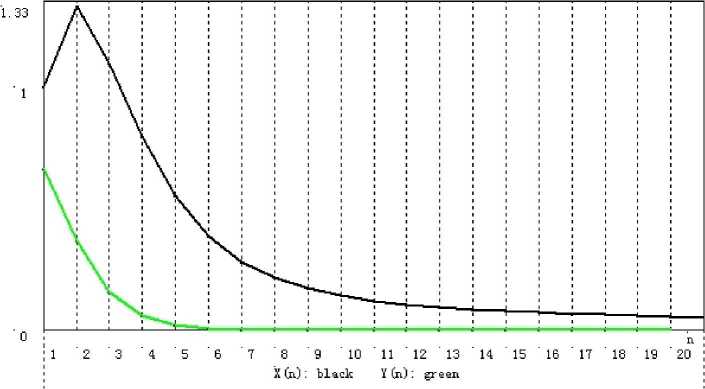
Convergence of $\{x_{n}\}$ and $\{y_{n}\}$ corresponding to Table 2

**Figure 3:**
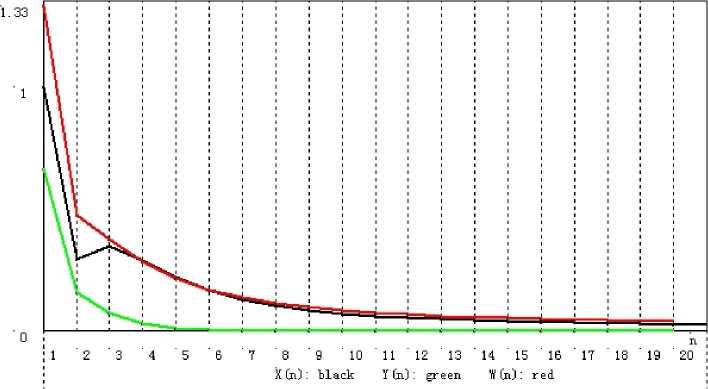
Convergence of $\{x_{n}\}$, $\{y_{n}\}$, and $\{w_{n}\}$ corresponding to Table 3

Do computational experiments on (3.10), (3.11), and (3.12) in Corollary 3.11. By using the codes of Visual Basic Six, we get Tables 1–3 and Figs. 1–3.

**Table 1:** Numerical results of $\{x_{n}\}$, $\{y_{n}\}$, and $\{w_{n}\}$ with initial $x_{1} = 1.0$ based on (3.10)

*n*	$x_{n}$	$y_{n}$	$w_{n}$
1	1.000000000000000	0.666666666666667	1.33333333333333
2	0.292893218813452	0.15857864376269	0.475735931288071
3	0.220137097256371	0.061496714509967	0.307483572549836
4	0.145846031275193	0.023285060663247	0.20956554596922
5	0.091822359822189	0.008843101812794	0.150332730817491
6	0.057343575321990	0.003446311415210	0.113728276701933
7	0.036498723210567	0.001390355550912	0.090373110809311
8	0.024079369242186	0.000580075366701	0.074829722304444
9	0.016612409147652	0.000248973723701	0.063986246991232
10	0.012011262300244	0.000109279280293	0.056060270790268
11	0.009075530986527	0.000048796789603	0.050016709342611
12	0.007124586938736	0.000022079062795	0.045239999667432
13	0.005771789196202	0.000010093356050	0.041352479737665
14	0.004794674685823	0.000004652013800	0.038113949064097
15	0.004062531254230	0.000002158417954	0.035365678169425
16	0.003496425067359	0.000001007010163	0.032998716038761
17	0.003047136222354	0.000000472032117	0.030935568833118
18	0.002682885282545	0.000000222161174	0.029119331499637
19	0.002382412236492	0.000000104930661	0.027507048057261
20	0.002130999790903	0.000000049715948	0.026065524753426

**Table 2:** Numerical results of $\{x_{n}\}$ and $\{y_{n}\}$ with initial $x_{1} = 1.0$ based on (3.11)

*n*	$x_{n}$	$y_{n}$
1	1.000000000000000	0.666666666666667
2	1.333333333333333	0.366666666666667
3	1.100000000000000	0.159259259259259
4	0.796296296296296	0.061546840958606
5	0.553921568627451	0.022846108140226
6	0.388383838383838	0.008539238539239
7	0.281794871794872	0.003291876082574
8	0.213971945367294	0.001318956985865
9	0.17014545117658	0.000548258406019
10	0.14090241034686	0.000235026741802
11	0.12056871854433	0.000103210253516
12	0.10579050985347	0.000046161543370
13	0.094585002365085	0.000020933489477
14	0.085764506388820	0.000009593718512
15	0.078601335767952	0.000004433092326
16	0.072636217763472	0.0000020619835782
17	0.067569139873525	0.0000009642921827
18	0.063196816788736	0.0000004530026220
19	0.059376412673457	0.0000002136378822
20	0.056004102629354	0.0000001010932937

**Table 3:** Numerical results of $\{x_{n}\}$, $\{y_{n}\}$, and $\{w_{n}\}$ with initial $x_{1} = 1.0$ based on (3.12)

*n*	$x_{n}$	$y_{n}$	$w_{n}$
1	1.000000000000000	0.66666666666667	1.333333333333333
2	0.292893218813452	0.15857864376269	0.475735931288071
3	0.347936514272221	0.07569664973395	0.378483248669752
4	0.290404855661242	0.03178852092125	0.286096688291246
5	0.221842355801224	0.01278310169095	0.217312728746085
6	0.167276133945363	0.00513758154788	0.169540191079954
7	0.12855725127519	0.00210398755141	0.136759190841874
8	0.101961142045651	0.00088311728422	0.113922129664937
9	0.083609952177013	0.00037957322278	0.097550318255454
10	0.070649802113264	0.00016648761182	0.085408144862541
11	0.061199308074553	0.00007423543142	0.07609131720753
12	0.054067201574777	0.00003353686476	0.068717035886434
13	0.048505907532604	0.00001530928652	0.062722146871100
14	0.044042300609567	0.00000704735258	0.057738959695359
15	0.040371134215207	0.00000326643477	0.053520533658321
16	0.037290303448763	0.00000152265596	0.049895913052360
17	0.034661992662647	0.00000071323249	0.046743117653451
18	0.032389319001134	0.00000033548179	0.043972605019241
19	0.030402139089697	0.00000015837395	0.041516938205506
20	0.028648273174303	0.00000007500477	0.039324174089535

### Remark 3.13

From Tables 1–3 and Figs. 1–3, we can see that for initial value $x_{1} = 1$, different choices of $x_{n+1}$ in $V_{n+1}$ lead to different rates of convergence. It is a natural phenomenon that the larger $x_{n+1}$ is chosen, the slower the rate of convergence is. Although $x_{n+1}$ in (3.11) is the slowest sequence among the three, it is worth being considered because of its “nice and simple” expression compared to the other two.

### Remark 3.14

Although both $x_{n+1}$ in (3.12) and (1.5) are chosen as the mid-point of $V_{n+1}$, they have different rates of convergence. From Table 1 in [8], we may find that the iterative sequence in (1.5) converges more rapidly than that in (3.12). From this point view, it is not easy for us to draw the conclusion which one is better, (1.3) or (3.1).
